# Supplementary material for: Development of chemokine network inhibitors using combinatorial saturation mutagenesis
Source: Commun Biol. 2025 Apr 3;8:549. doi: 10.1038/s42003-025-07778-6 (PMC11969024; doi:10.1038/s42003-025-07778-6)
Supplement: Supplementary file 1 — Supplementary Information [file 42003_2025_7778_MOESM1_ESM.pdf]

Supplementary Information: Figures and Tables
Development of chemokine network inhibitors using combinatorial saturation mutagenesis

Jhanna Kryukova, Serena Vales, Megan Payne, Gintare Smagurauskaite, Soumyanetra Chandra, Charlie J. Clark, Graham Davies, and Shoumo Bhattacharya

Table of contents
Figures
Supplementary Figure 1. Impact of HD2 single mutations on chemotaxis of THP1 cells
Supplementary Figure 2. Impact of HD2 single mutations on chemotaxis of activated T-cells.
Supplementary Figure 3. Impact of HD2 single mutations on chemotaxis of Jurkat:CXCR1 cells.
Supplementary Figure 4. Impact of HD2 single and combinatorial mutations on chemotaxis of THP1 cells.
Supplementary Figure 5. Impact of HD2 single and combinatorial mutations on chemotaxis of activated T-cells.
Supplementary Figure 6. Chemokine expression in selected inflammatory diseases.
Supplementary Figure 7. Impact of HD2 single and combinatorial mutations on chemotaxis induced by a pool of synthetic chemokines known to be expressed in atherosclerotic plaque.
Supplementary Figure 8. Impact of HD2 single and combinatorial mutations on chemotaxis induced by a pool of synthetic chemokines known to be expressed in cytokine-stimulated pancreatic islets
Supplementary Figure 9. Impact of HD2 single and combinatorial mutations on chemotaxis induced by a pool of synthetic chemokines known to be expressed in rheumatoid arthritis synovial tissue.
Supplementary Figure 10. Representative dose-response curves showing effect of indicated human chemokine pools on ATC (activated T-cell) migration.
Supplementary Figure 11. Representative dose-response curves showing effect of indicated human chemokine pools on THP1 cell migration.
Supplementary Figure 12. Schematic of mutant phage library generation and parallel phage display assay setup.
Supplementary Figure 13. White blood cell identification using flow cytometry.
Supplementary Figure 14. Representative dose-response curves showing effect of indicated human CC class chemokines on THP1 or ATC (activated T-cell) migration.
Supplementary Figure 15. Representative dose-response curves showing effect of indicated human CXC-class chemokines on J:CXCR or ATC (activated T-cell) migration.
Supplementary Figure 16. Representative dose-response curves showing effect of indicated human chemokine pools on THP1 or ATC (activated T-cell) migration.
Tables
Supplementary Table 1. Mean and standard error (SE) of chemokine pEC50 and pEC80 values
Supplementary Table 2. Mean and standard error (SE) of peptide IC50 values
Supplementary Table 3. Human Biotinylated Bait Panel
Supplementary Table 4. Chemokine Suppliers for Cell Migration Experiments
Supplementary Table 5. Plaque Atherosclerosis Chemokine Pool Construction
Supplementary Table 6. Islet Cytokine Stimulated Chemokine Pool Construction
Supplementary Table 7. Synovium Rheumatoid Chemokine Pool Construction
Supplementary Table 8. Peptide Sequences
Supplementary Table 9a. Exact P values for phage-display mutagenesis analyses, comparisons to control
Supplementary Table 9b. Exact P values for cell migration experiments, comparisons to control
Supplementary Table 9c. Exact P values for cell migration experiments, comparisons to control
Supplementary Table 9d. Exact P values for cell migration experiments, comparisons to control
Supplementary Table 9e. Exact P values for pIC50 comparisons to control
Supplementary Table 9f. Exact P values for Arpeggio bond numbers, comparisons to control
Supplementary Table 9g. Exact P values for cell migration experiments, comparisons to control

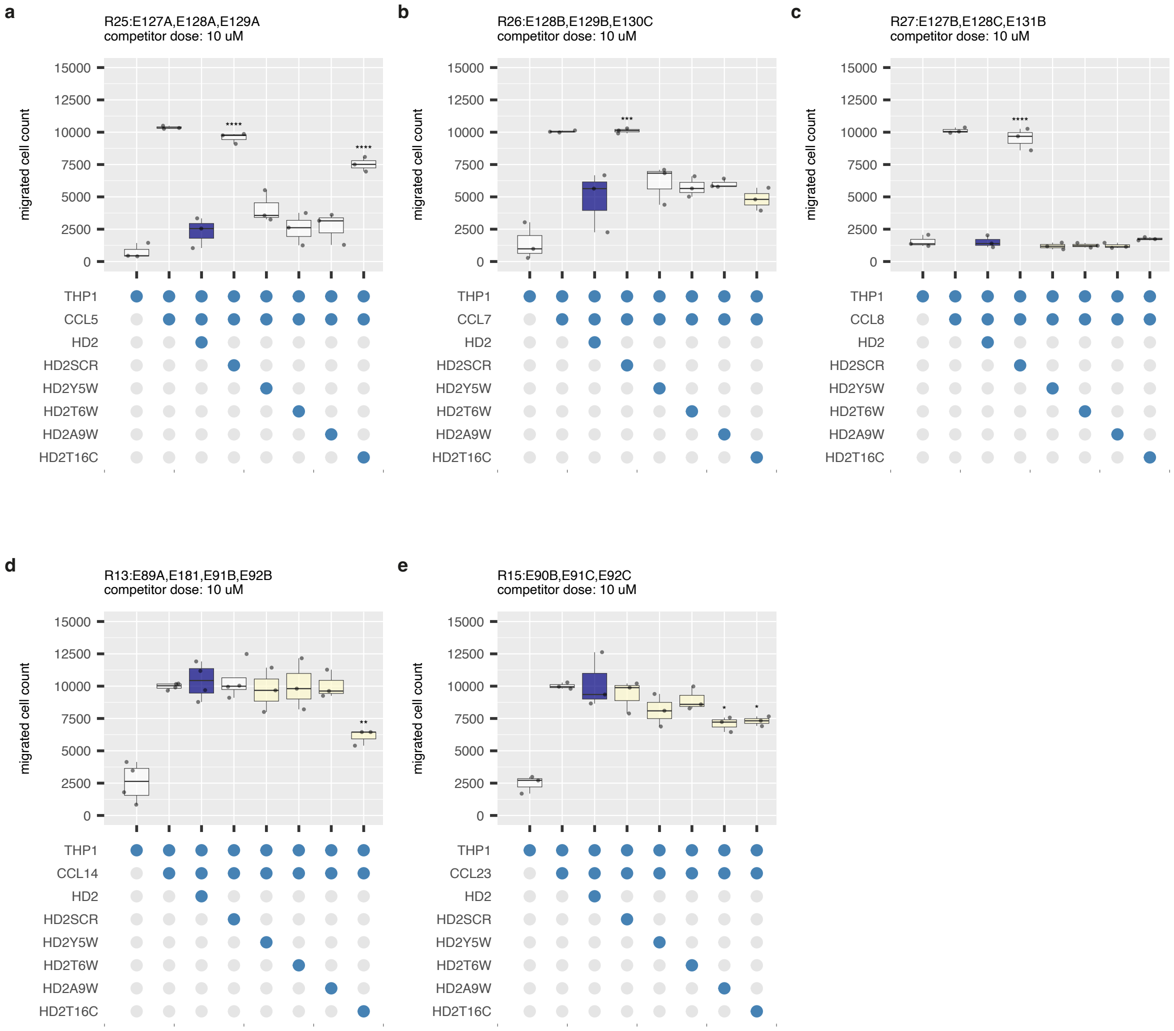

Supplementary Figure 1. Impact of HD2 single mutations on chemotaxis of THP1 cells. a-e, Box-whisker plots showing the effect of indicated peptides on migration of THP1 monocyte cells induced by indicated human chemokines. Each plot shows the median as centre, 25th and 75th percentile as bounds, and 1.5*interquartile range as whiskers. All experiments were performed as three technical and at least three biological replicates. Individual biological replicate data points (mean of technical replicates) are shown. Y-axis in each panel shows cell count normalized to the median value of migrated cells in the presence of chemokine alone, set at 10000 cells. X-axis shows constituents of each experiment as blue-filled dots. Chemokine and cell type names are indicated. SCR is a scrambled version of HD2. Peptide concentrations are indicated in each figure. Chemokines were at EC80 doses. Statistically significant differences (compared to control, n = minimum 3 biologically independent experiments per group), were identified using a two-sided Dunnett's test with correction for multiple comparisons and are indicated by asterisks: **** $P \leq 0.0001$, *** $P \leq 0.001$, ** $P \leq 0.01$. The control box in each panel is coloured blue, while boxes showing a negative value for difference from control (identified from Dunnett's test) are shown as yellow. Exact P-values and numbers of biologically independent experiments per group are provided in Supplementary Table 1g. R numbers in the title indicate replicate ID, E numbers indicate individual experiments.

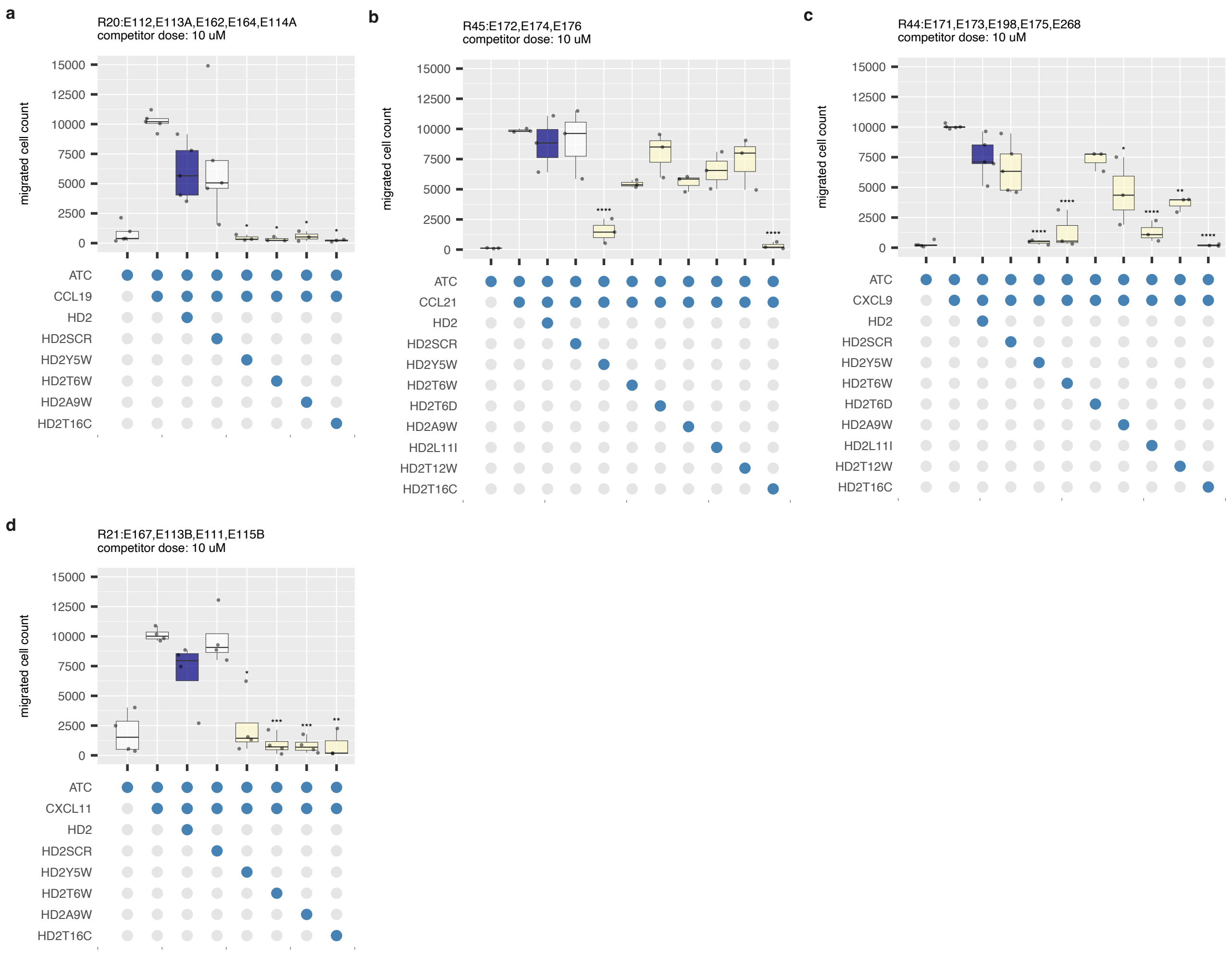

Supplementary Figure 2. Impact of HD2 single mutations on chemotaxis of activated T-cells. A-d, Box-whisker plots showing the effect of indicated peptides on migration of ATC (activated T-cells) induced by indicated human chemokines. Each plot shows the median as centre, 25th and 75th percentile as bounds, and 1.5*interquartile range as whiskers. All experiments were performed as three technical and at least three biological replicates. Individual biological replicate data points (mean of technical replicates) are shown. Y-axis in each panel shows cell count normalized to the median value of migrated cells in the presence of chemokine alone, set at 10000 cells. X-axis shows constituents of each experiment as blue-filled dots. Chemokine and cell type names are indicated. SCR is a scrambled version of HD2. Peptide concentrations are indicated in each figure. Chemokines were at EC80 doses. Statistically significant differences (compared to control, n = 3 biologically independent experiments per group), were identified using a two-sided Dunnett's test with correction for multiple comparisons and are indicated by asterisks: **** $P \leq 0.0001$, *** $P \leq 0.001$, ** $P \leq 0.01$, * $P \leq 0.05$. The control box in each panel is coloured blue, while boxes showing a negative value for difference from control (identified from Dunnett's test) shown as yellow. Numbers of biologically independent experiments per group and exact P-values are provided in Supplementary Table 1g. R numbers indicate replicate ID, E numbers indicate individual experiments.

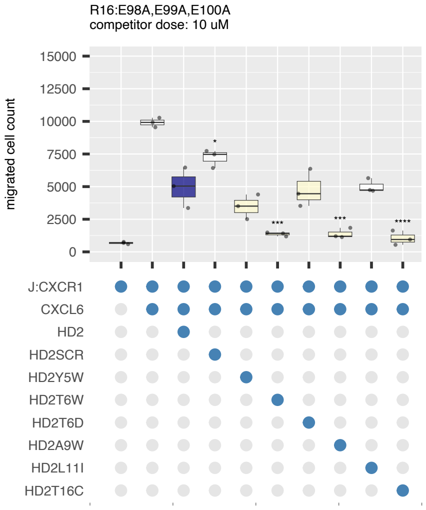

Supplementary Figure 3. Impact of HD2 single mutations on chemotaxis of Jurkat:CXCR1 cells. Box-whisker plot showing the effect of indicated peptides on migration of J:CXCR1 cells (Jurkat cells stably transfected with CXCR1) induced by human CXCL6. Each plot shows the median as centre, 25th and 75th percentile as bounds, and 1.5*interquartile range as whiskers. The experiment was performed as three technical and three biological replicates. Individual biological replicate data points (mean of technical replicates) are shown. Y-axis in each panel shows cell count normalized to the median value of migrated cells in the presence of chemokine alone, set at 10000 cells. X-axis shows constituents of each experiment as blue-filled dots. SCR is a scrambled version of HD2. Peptide concentrations are indicated in each figure. Chemokines were at EC80 doses. Statistically significant differences (compared to control, n = 3 biologically independent experiments per group), were identified using a two-sided Dunnett's test with correction for multiple comparisons and are indicated by asterisks: *** $P \leq 0.001$, ** $P \leq 0.01$, * $P \leq 0.05$. The control box in each panel is coloured blue, while boxes showing a negative value for difference from control (identified from Dunnett's test) are shown as yellow. Exact P-values and numbers of biologically independent experiments per group are provided in Supplementary Table 1g. R numbers in the title indicate replicate ID, E numbers indicate individual experiments.

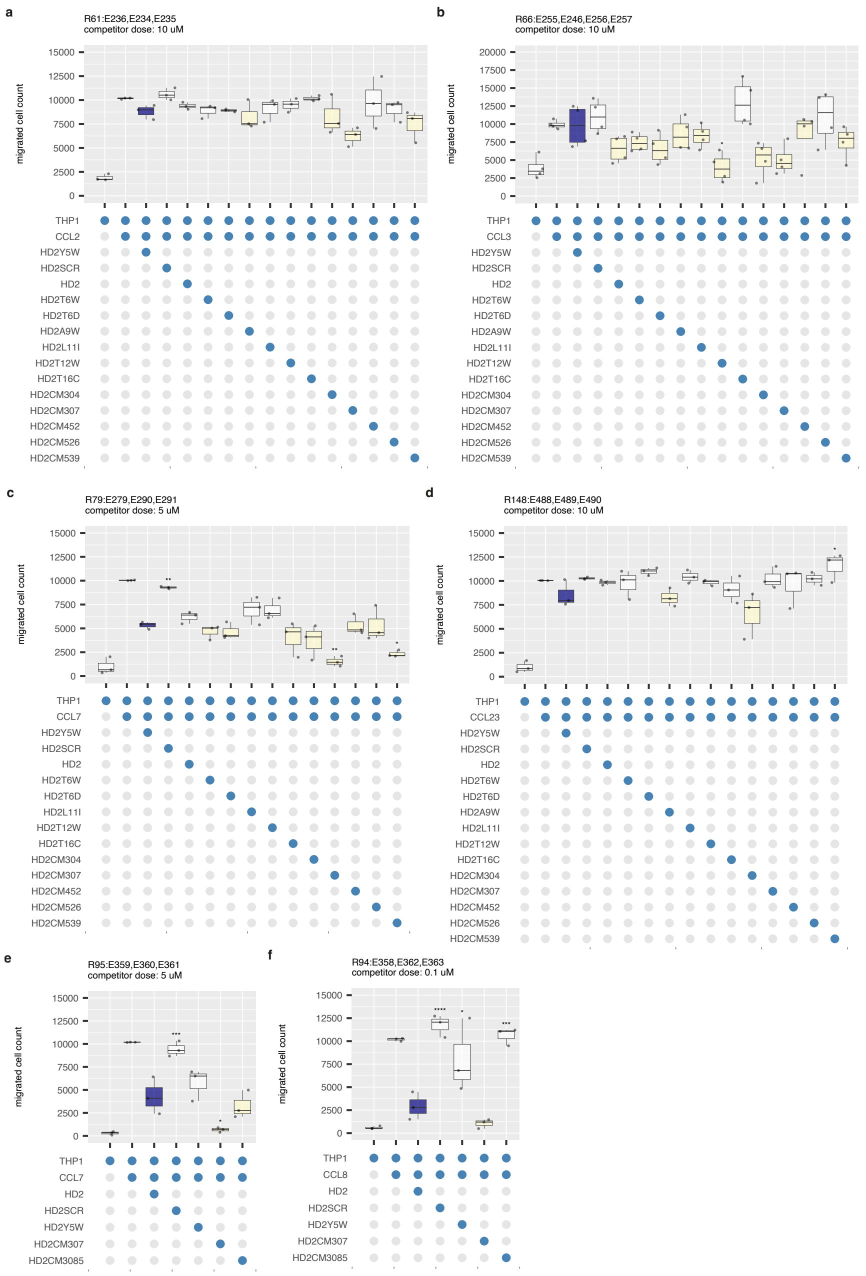

Supplementary Figure 4. Impact of HD2 single and combinatorial mutations on chemotaxis of THP1 cells. a-e, Box-whisker plots showing the effect of indicated peptides on migration of THP1 monocyte cells induced by indicated human chemokines. Each plot shows the median as centre, 25th and 75th percentile as bounds, and 1.5*interquartile range as whiskers. All experiments were performed as three technical and at least three biological replicates. Individual biological replicate data points (mean of technical replicates) are shown. Y-axis in each panel shows cell count normalized to the median value of migrated cells in the presence of chemokine alone, set at 10000 cells. X-axis shows constituents of each experiment as blue-filled dots. Chemokine and cell type names are indicated. SCR is a scrambled version of HD2. Peptide concentrations are indicated in each figure. Chemokines were at EC80 doses. Statistically significant differences (compared to control, n = minimum 3 biologically independent experiments per group), were identified using a two-sided Dunnett's test with correction for multiple comparisons and are indicated by asterisks: **** $P \leq 0.0001$, *** $P \leq 0.001$, ** $P \leq 0.01$, * $P \leq 0.05$.The control box in each panel is coloured blue, while boxes showing a negative value for difference from control (identified from Dunnett's test) are shown as yellow. Exact P-values and numbers of biologically independent experiments per group are provided in Supplementary Table 1g. R numbers in the title indicate replicate ID, E numbers indicate individual experiments.

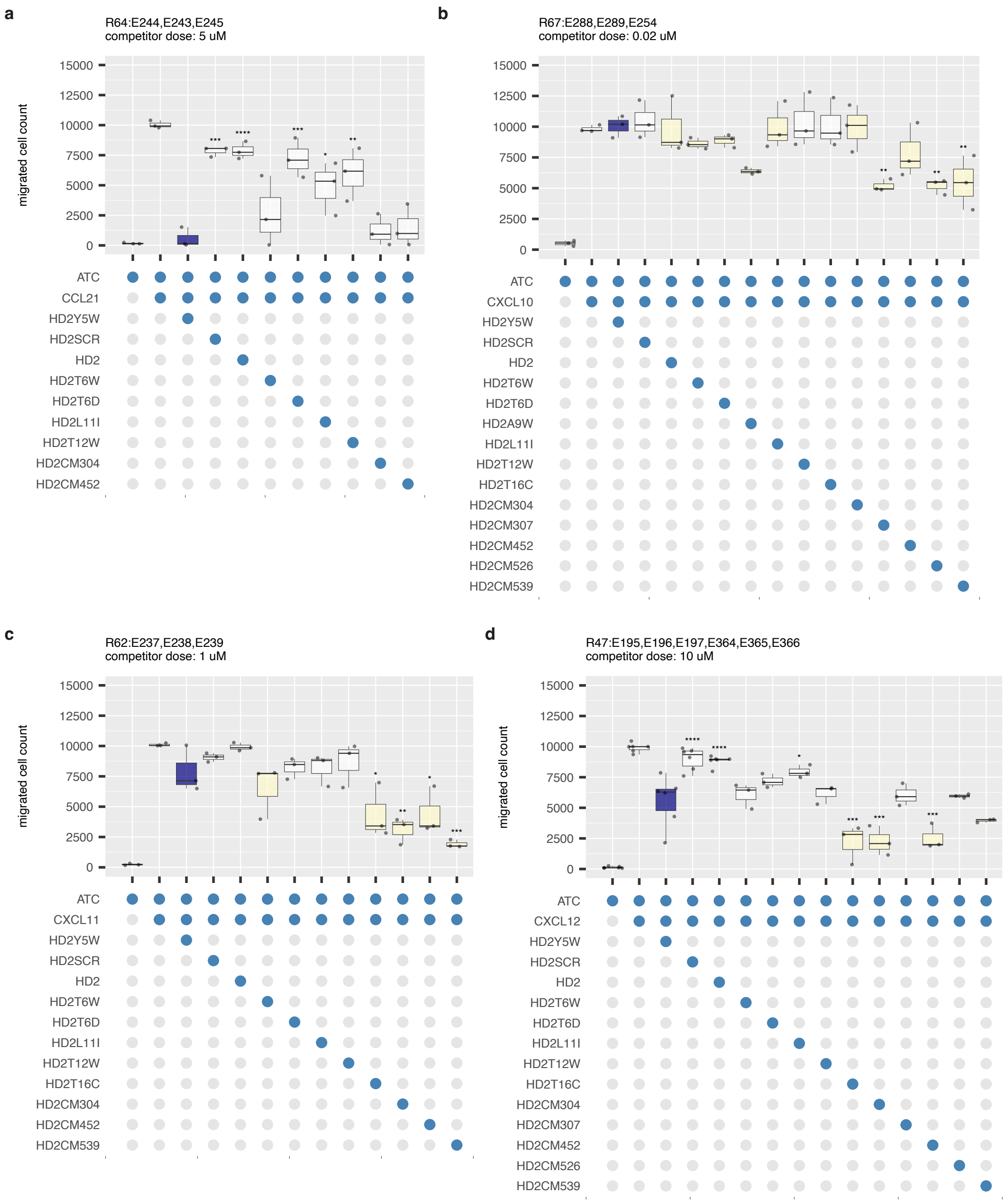

Supplementary Figure 5. Impact of HD2 single and combinatorial mutations on chemotaxis of activated T-cells. a-c, Box-whisker plots showing the effect of indicated peptides on migration of activated T-cells (ATC) cells induced by indicated human chemokines. Each plot shows the median as centre, 25th and 75th percentile as bounds, and 1.5*interquartile range as whiskers. All experiments were performed as three technical and at least three biological replicates, and individual biological replicate data points (mean of technical replicates) are shown. Y-axis in each panel shows cell count normalized to the median value of migrated cells in the presence of chemokine alone, set at 10000 cells. X-axis shows constituents of each experiment as blue-filled dots. Chemokine and cell type names are indicated. SCR is a scrambled version of HD2. Peptide concentrations are indicated in each figure. Chemokines were at EC80 doses. Statistically significant differences (compared to control, n = minimum 3 biologically independent experiments per group), were identified using a two-sided Dunnett's test with correction for multiple comparisons and are indicated by asterisks: **** P ≤ 0.0001, *** P ≤ 0.001, ** P ≤ 0.01, * P ≤ 0.05. The control box in each panel is coloured blue, while boxes showing a negative value for difference from control (identified from Dunnett's test) are shown as yellow. Exact P-values and numbers of biologically independent experiments per group are provided in Supplementary Table 1g. R numbers in the title indicate replicate ID, E numbers indicate individual experiments.

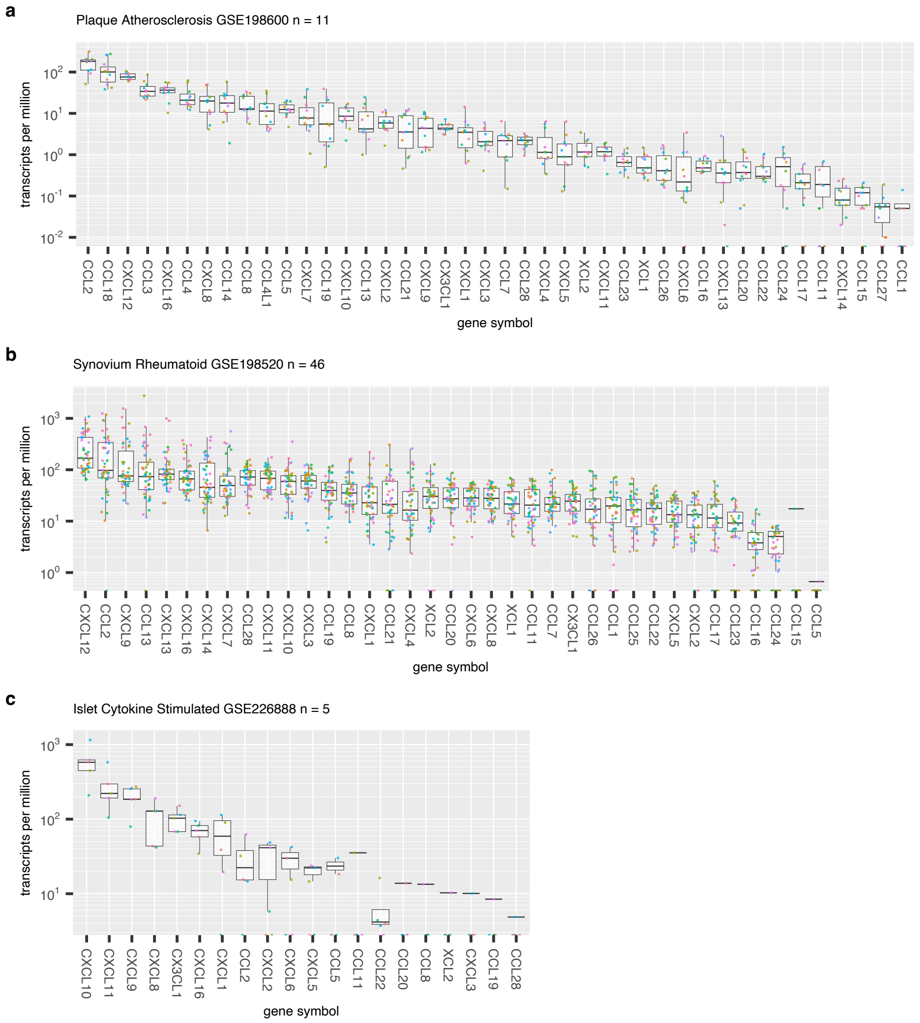

Supplementary Figure 6. Chemokine expression in selected inflammatory diseases. Box-whisker plots showing chemokines in **a,** atherosclerotic plaque, **b,** rheumatoid synovium, and **c,** cytokine-stimulated pancreatic donor islets. Each plot shows the median as centre, 25th and 75th percentile as bounds, and 1.5*interquartile range as whiskers. Y-axis indicates transcript count per million, X-axis the chemokine gene symbol. GEO database source identity (GSE number) is indicated in the titles, as is n, the number of samples. Individual datapoints are shown.

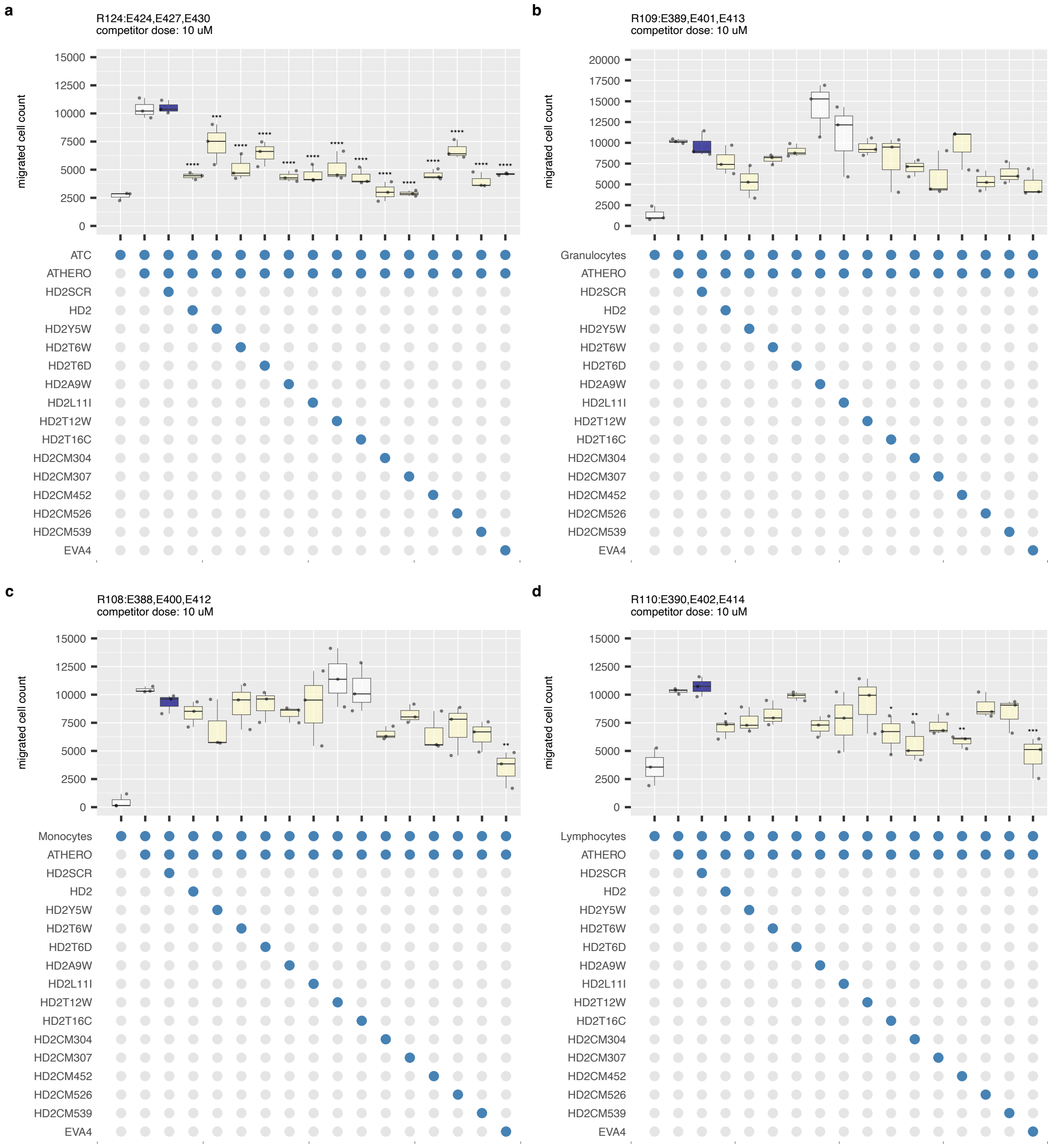

Supplementary Figure 7. Impact of HD2 single and combinatorial mutations on chemotaxis induced by a pool of synthetic chemokines known to be expressed in atherosclerotic plaque. a-d, Box-whisker plots showing the effect of indicated peptides on migration of indicated cells induced by a chemokine pool "ATHERO" designed based on expression levels in human atherosclerotic plaque. Each plot shows the median as centre, 25th and 75th percentile as bounds, and 1.5*interquartile range as whiskers. All experiments were performed as three technical and at least three biological replicates, and individual biological replicate data points (mean of technical replicates) are shown. Y-axis in each panel shows cell count normalized to the median value of migrated cells in the presence of chemokine alone, set at 10000 cells. X-axis shows constituents of each experiment as blue-filled dots. Chemokine pool and cell type names are indicated. SCR is a scrambled version of HD2. Peptide concentrations are indicated in each figure. The chemokine pool was at its EC50 dose. Statistically significant differences (compared to control, n = minimum 3 biologically independent experiments per group), were identified using a two-sided Dunnett's test with correction for multiple comparisons and are indicated by asterisks: **** $P \leq 0.0001$, *** $P \leq 0.001$, ** $P \leq 0.01$, * $P<0.05$. The control box in each panel is coloured blue, while boxes showing a negative value for difference from control (identified from Dunnett's test) are shown as yellow. Exact P-values and numbers of numbers of biologically independent experiments per group are provided in Supplementary Table 1g. R numbers in the title indicate replicate ID, E numbers indicate individual experiments.

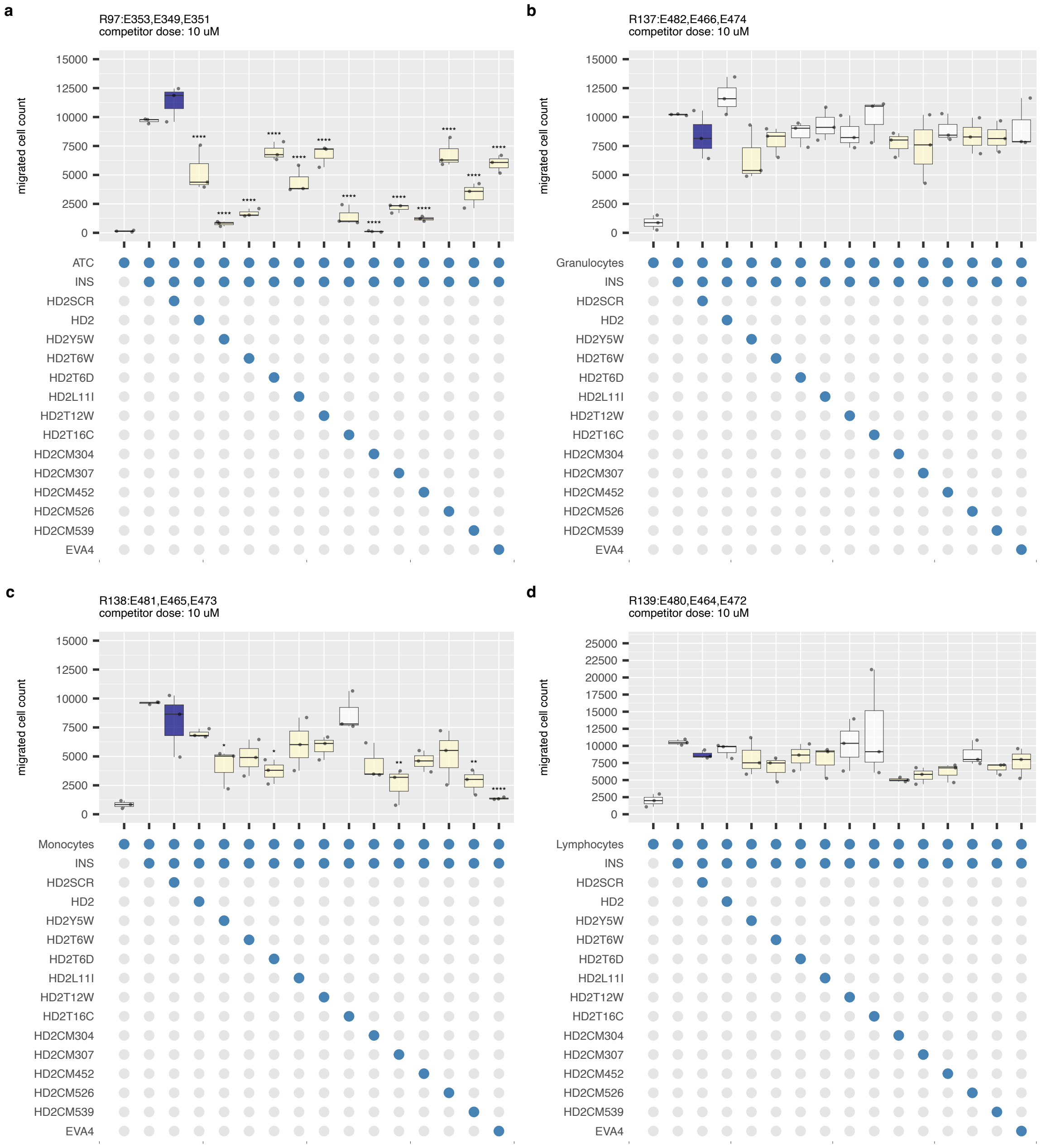

Supplementary Figure 8. Impact of HD2 single and combinatorial mutations on chemotaxis induced by a pool of synthetic chemokines known to be expressed in cytokine-stimulated pancreatic islets. a-d, Box-whisker plots showing the effect of indicated peptides on migration of indicated cells induced by a chemokine pool "INS" designed based on expression levels in cytokine-stimulated pancreatic islets. Each plot shows the median as centre, 25th and 75th percentile as bounds, and 1.5*interquartile range as whiskers. All experiments were performed as three technical and at least three biological replicates, and individual biological replicate data points (mean of technical replicates) are shown. Y-axis in each panel shows cell count normalized to the median value of migrated cells in the presence of chemokine alone, set at 10000 cells. X-axis shows constituents of each experiment as blue-filled dots. Chemokine pool and cell type names are indicated. SCR is a scrambled version of HD2. Peptide concentrations are indicated in each figure. The chemokine pool was at its EC50 dose. Statistically significant differences (compared to control, n = minimum 3 biologically independent experiments per group), were identified using a two-sided Dunnett's test with correction for multiple comparisons and are indicated by asterisks: **** $P \leq 0.0001$, *** $P \leq 0.001$, ** $P \leq 0.01$, * $P<0.05$. The control box in each panel is coloured blue, while boxes showing a negative value for difference from control (identified from Dunnett's test) are shown as yellow. Exact P-values and numbers of biologically independent experiments per group are provided in Supplementary Table 1g. R numbers in the title indicate replicate ID, E numbers indicate individual experiments.

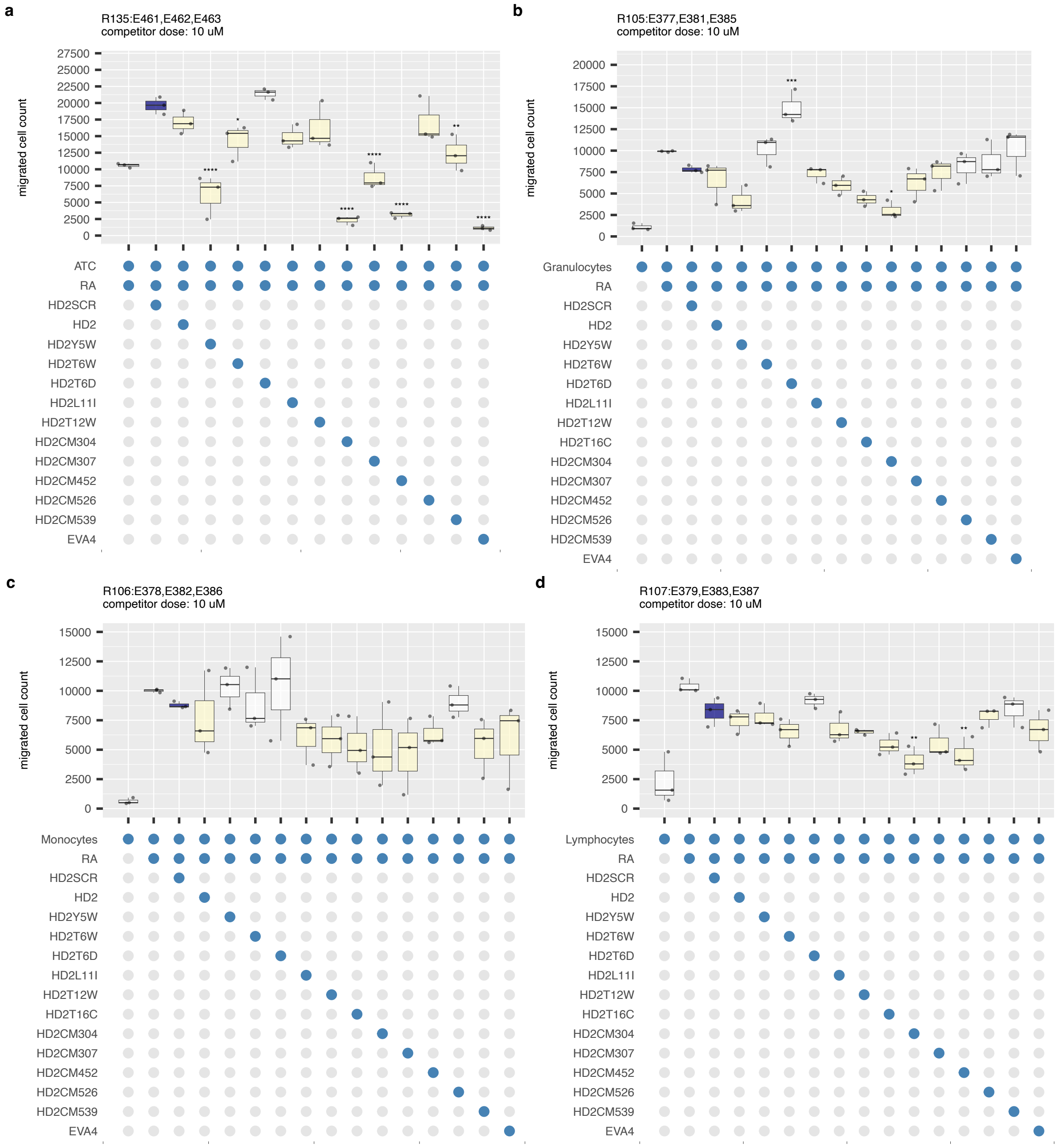

Supplementary Figure 9. Impact of HD2 single and combinatorial mutations on chemotaxis induced by a pool of synthetic chemokines known to be expressed in rheumatoid arthritis synovial tissue. a-d, Box-whisker plots showing the effect of indicated peptides on migration of indicated cells induced by a chemokine pool "RA" designed based on expression levels in rheumatoid arthritis synovial tissue. Each plot shows the median as centre, 25th and 75th percentile as bounds, and 1.5*interquartile range as whiskers. All experiments were performed as three technical and at least three biological replicates, and individual biological replicate data points (mean of technical replicates) are shown. Y-axis in each panel shows cell count normalized to the median value of migrated cells in the presence of chemokine alone, set at 10000 cells. X-axis shows constituents of each experiment as blue-filled dots. Chemokine pool and cell type names are indicated. SCR is a scrambled version of HD2. Peptide concentrations are indicated in each figure. The chemokine pool was at its EC50 dose. Statistically significant differences (compared to control, n = minimum 3 biologically independent experiments per group), were identified using a two-sided Dunnett's test with correction for multiple comparisons and are indicated by asterisks: **** $P \leq 0.0001$, *** $P \leq 0.001$, ** $P \leq 0.01$, * $P<0.05$. The control box in each panel is coloured blue, while boxes showing a negative value for difference from control (identified from Dunnett's test) are shown as yellow. Exact P-values and numbers of biologically independent experiments per group are provided in Supplementary Table 1g. R numbers in the title indicate replicate ID, E numbers indicate individual experiments.

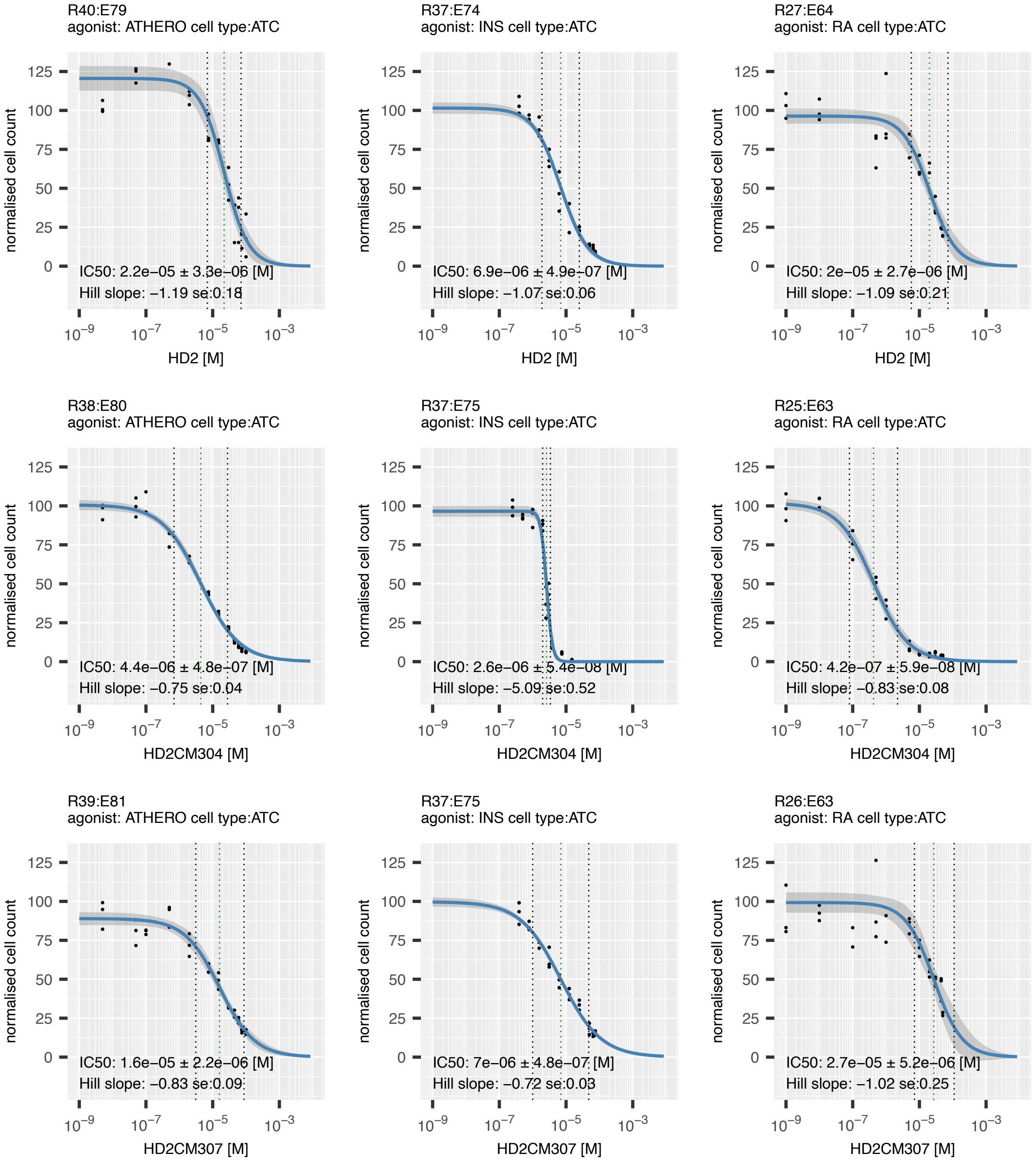

Supplementary Figure 10. Representative dose-response curves showing effect of indicated human chemokine pools on ATC (activated T-cell) migration. Y-axis shows percent migrated cells normalized to maximum migration (set at 100%). X-axis shows inhibitor concentration (molar). Technical replicates at each inhibitor concentration (n=3) are shown as individual data points. The dose-response curves (solid blue lines) and 95% confidence intervals (grey ribbons) were calculated using a 3-parameter log-logistic plot, setting the maximum response to zero. Dotted green lines indicate IC50 and dotted black lines IC20 and IC80. The Hill slope and IC50(M, estimated from the X-intercept) ± standard error (se) of the estimate is indicated in each plot. E numbers indicate individual experiments.

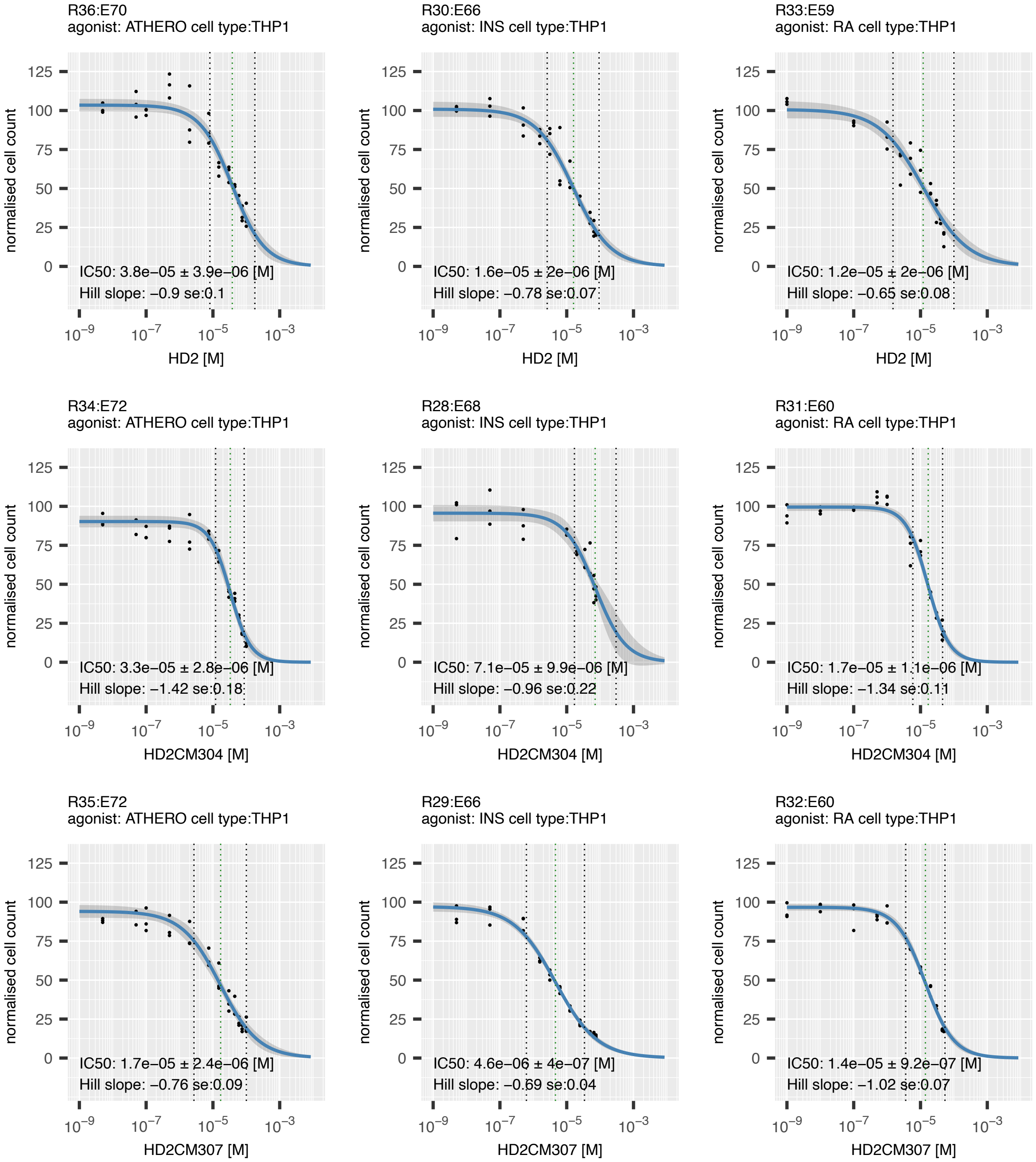

Supplementary Figure 11. Representative dose-response curves showing effect of indicated human chemokine pools on THP1 cell migration. Y-axis shows percent migrated cells normalized to maximum migration (set at 100%). X-axis shows inhibitor concentration (molar). Technical replicates at each inhibitor concentration (n=3) are shown as individual data points. The dose-response curves (solid blue lines) and 95% confidence intervals (grey ribbons) were calculated using a 3-parameter log-logistic plot, setting the maximum response to zero. Dotted green lines indicate IC50 and dotted black lines IC20 and IC80. The Hill slope and IC50(M, estimated from the X-intercept) ± standard error (se) of the estimate is indicated in each plot. E numbers indicate individual experiments.

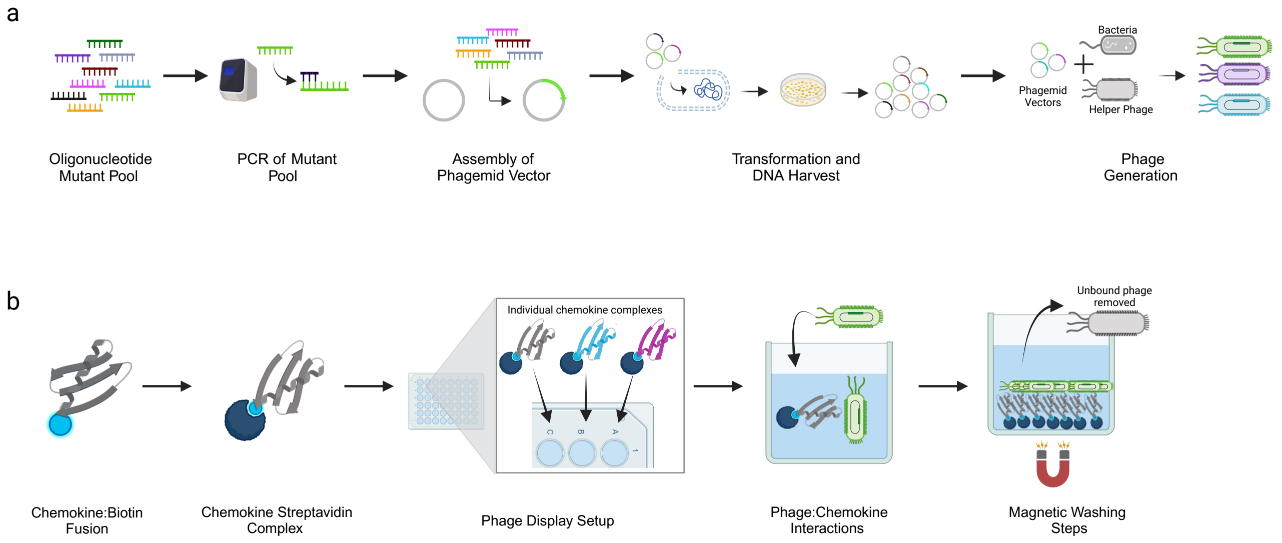

Supplementary Figure 12. Schematic of mutant phage library generation and parallel phage display assay setup. **a,** For saturation mutagenesis, oligonucleotide pools were designed to encode the HD2 sequence, the HD2 SCR sequence and all single mutant HD2 peptides, mutated at each residue with the NNK codon. For combinatorial mutagenesis, oligonucleotide pools were designed to encode for the HD2 sequence, the HD2 SCR sequence, top-hit single mutants and combinations of the top-hit mutant peptides. Oligonucleotide pools were purchased from Genscript. The oligonucleotide pools were first amplified by PCR and cloned into the prSTOP4 phagemid vector. Transformation into E. coli and subsequent DNA harvest was completed to cover all desired peptide sequences within the input library. Phage was produced by transforming of the phagemid vectors containing the peptide sequences of interest into E. coli supplemented with helper phage. Phage libraries were then analysed by next-generation sequencing to confirm the desired library heterogeneity. **b,** Parallel phage display assays were set up as described in methods. Biotinylated chemokines were incubated with streptavidin-coated magnetic nanobeads and then transferred to individual wells of a 96-well plate and allowed to bind overnight. Phage library was then added, and allowed to bind to the chemokine-streptavidoin-bead complex. Beads were washed while being retained in the well using a magnet, to remove unbound phage. Further details regarding methodology can be found in methods. Created in BioRender. Bhattacharya, S. (2025) https://BioRender.com/h59y819.

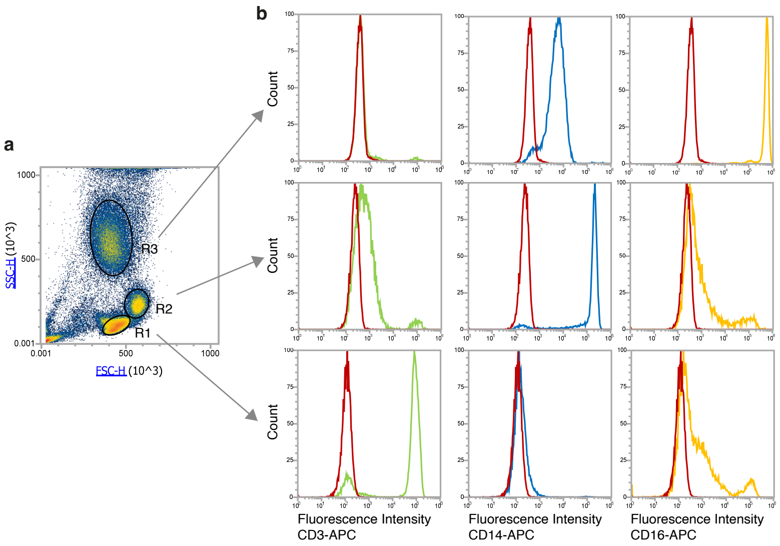

Supplementary Figure 13. White blood cell identification using flow cytometry. a, White blood cell gating into three regions (R1, R2 and R3) drawn around three distinct cell populations. X-axis shows forward scatter height (FSC-H) ,and Y-axis shows side-scatter height (SSC-H). **b,** Histograms of cells from respective regions plotted against fluorescence intensity for anti-CD3-APC (green, T-lymphocytes), anti-CD14-APC (blue, monocytes), and anti-CD16-APC (yellow, granulocytes). Red histogram in each panel indicates negative control stained with anti- IgG1. X-axis shows cell count normalised to the mode. Y-axis shows fluorescent intensity.

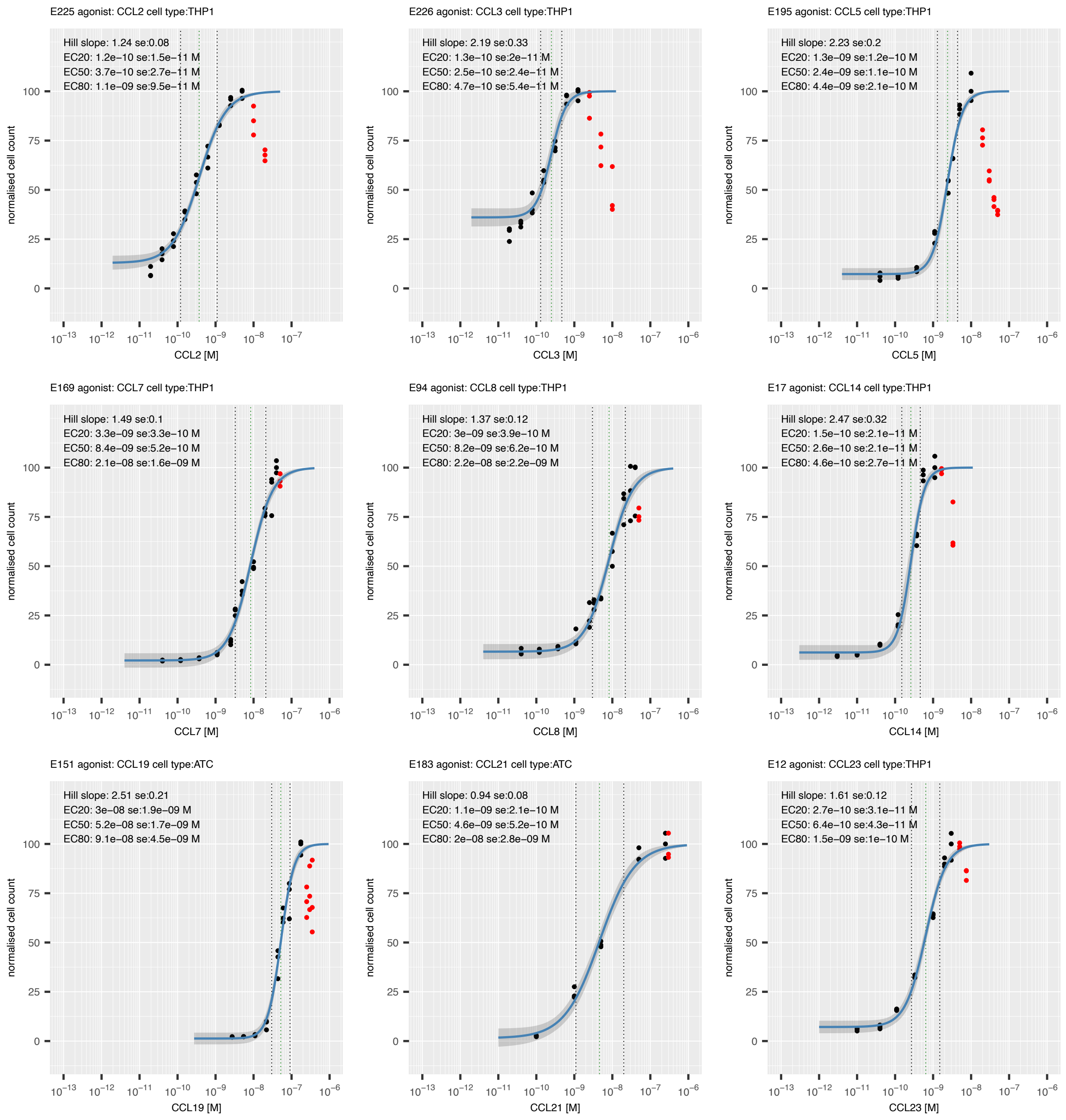

Supplementary Figure 14. Representative dose-response curves showing effect of indicated human CC class chemokines on THP1 or ATC (activated T-cell) migration. Y-axis shows percent migrated cells normalized to maximum migration (set at 100%). X-axis shows inhibitor concentration (molar). Technical replicates at each inhibitor concentration (n=3) are shown as individual data points. The dose-response curves (solid blue lines) and 95% confidence intervals (grey ribbons) were calculated using a 3-parameter log-logistic plot, setting the maximum response to 100%. Dotted green lines indicate EC50 and dotted black lines EC20 and EC80. The Hill slope, EC20, EC50 and EC80 (M, estimated from the X-intercept) ± standard error (se) of the estimate is indicated in each plot. Data points showing a reduction from the peak response are indicated in red and were excluded from curve-fitting analyses. E numbers indicate individual experiments.

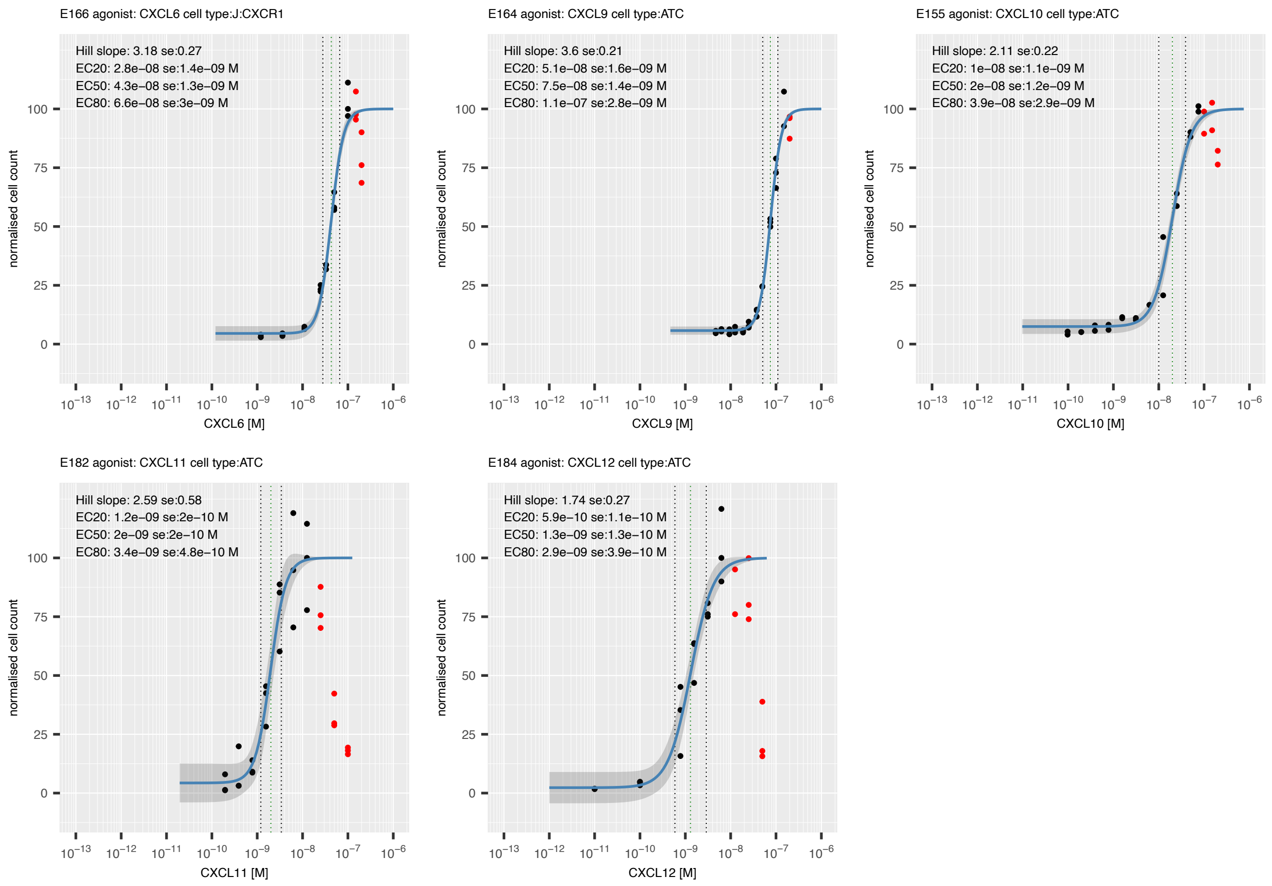

Supplementary Figure 15. Representative dose-response curves showing effect of indicated human CXC-class chemokines on J:CXCR or ATC (activated T-cell) migration. Y-axis shows percent migrated cells normalized to maximum migration (set at 100%). X-axis shows inhibitor concentration (molar). Technical replicates at each inhibitor concentration (n=3) are shown as individual data points. The dose-response curves (solid blue lines) and 95% confidence intervals (grey ribbons) were calculated using a 3-parameter log-logistic plot, setting the maximum response to 100%. Dotted green lines indicate EC50 and dotted black lines EC20 and EC80. The Hill slope, EC20, EC50 and EC80 (M, estimated from the X-intercept) ± standard error (se) of the estimate is indicated in each plot. Data points showing a reduction from the peak response are indicated in red and were excluded from curve-fitting analyses. E numbers indicate individual experiments.

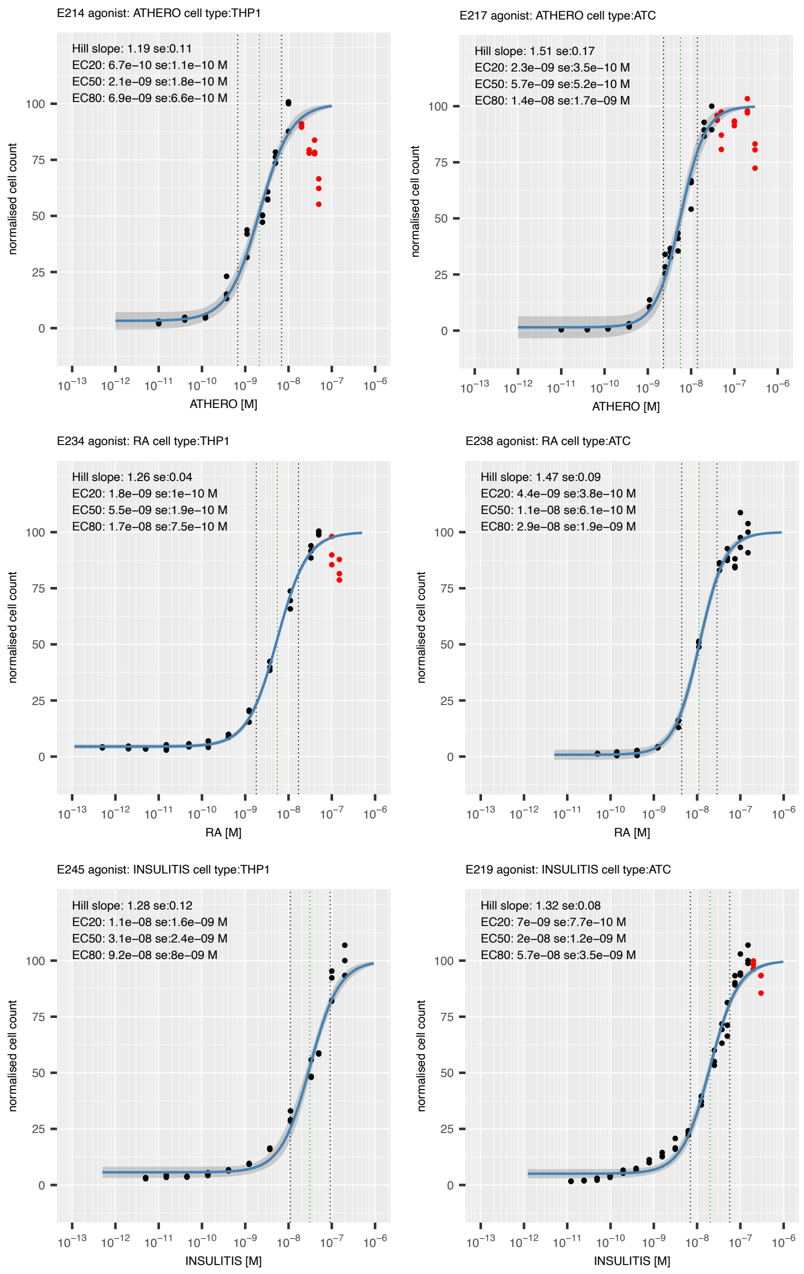

Supplementary Figure 16. Representative dose-response curves showing effect of indicated human chemokine pools on THP1 or ATC (activated T-cell) migration. Y-axis shows percent migrated cells normalized to maximum migration (set at 100%). X-axis shows inhibitor concentration (molar). Technical replicates at each inhibitor concentration (n=3) are shown as individual data points. The dose-response curves (solid blue lines) and 95% confidence intervals (grey ribbons) were calculated using a 3-parameter log-logistic plot, setting the maximum response to 100%. Dotted green lines indicate EC50 and dotted black lines EC20 and EC80. The Hill slope, EC20, EC50 and EC80 (M, estimated from the X-intercept) ± standard error (se) of the estimate is indicated in each plot. Data points showing a reduction from the peak response are indicated in red and were excluded from curve-fitting analyses. E numbers indicate individual experiments.

Supplementary Table 1. Mean and standard error (SE) of chemokine pEC50 and pEC80 values

Chemokine	Cells	Replicates	Mean pEC80 [M]	SE pEC80 [M]	Mean pEC50 [M]	SE pEC50 [M]
CCL2	THP1	3	8.35	0.51	8.73	0.51
CCL3	THP1	2	9.31	0.02	9.64	0.02
CCL5	THP1	7	8.36	0.03	8.65	0.03
CCL7	THP1	4	8.02	0.12	8.37	0.12
CCL8	THP1	7	7.84	0.04	8.19	0.04
CCL14	THP1	4	9.23	0.04	9.61	0.04
CCL19	ATC	6	7.64	0.27	7.91	0.27
CCL21	ATC	3	7.42	0.20	7.98	0.20
CCL23	THP1	3	8.66	0.11	8.97	0.11
CXCL6	J:CXCR1	7	7.45	0.08	7.71	0.08
CXCL9	ATC	5	7.22	0.19	7.40	0.19
CXCL10	ATC	3	7.50	0.05	7.87	0.05
CXCL11	ATC	3	7.99	0.52	8.25	0.52
CXCL12	ATC	3	8.54	0.05	8.93	0.05
ATHERO	THP1	4	8.29	0.04	8.73	0.04
ATHERO	ATC	5	7.39	0.18	7.84	0.18
INSULITIS	THP1	4	7.22	0.08	7.75	0.08
INSULITIS	ATC	3	7.17	0.04	7.53	0.04
RA	THP1	4	7.72	0.06	8.20	0.06
RA	ATC	3	7.42	0.06	7.87	0.06

Supplementary Table 2. Mean and standard error (SE) of peptide IC50 values

Chemokine	Peptide	Cells	Replicates	Mean pIC50 [M]	SE pIC50 [M]	Mean IC50 [M]
ATHERO	HD2CM304	ATC	3	5.43	0.14	3.7e-06
ATHERO	HD2	ATC	3	4.82	0.14	1.5e-05
ATHERO	HD2CM307	ATC	3	4.61	0.15	2.5e-05
ATHERO	HD2CM307	THP1	3	4.84	0.15	1.4e-05
ATHERO	HD2CM304	THP1	3	4.44	0.04	3.6e-05
ATHERO	HD2	THP1	3	4.39	0.05	4.1e-05
INS	HD2CM304	ATC	3	5.72	0.12	1.9e-06
INS	HD2CM307	ATC	3	5.11	0.14	7.8e-06
INS	HD2	ATC	3	4.88	0.26	1.3e-05
INS	HD2CM307	THP1	3	5.02	0.17	9.5e-06
INS	HD2	THP1	3	4.54	0.14	2.9e-05
INS	HD2CM304	THP1	3	4.23	0.05	5.9e-05
RA	HD2CM304	ATC	3	6.35	0.01	4.5e-07
RA	HD2	ATC	3	5.02	0.18	9.5e-06
RA	HD2CM307	ATC	3	4.74	0.24	1.8e-05
RA	HD2CM307	THP1	3	4.95	0.08	1.1e-05
RA	HD2CM304	THP1	3	4.77	0.13	1.7e-05
RA	HD2	THP1	3	4.56	0.19	2.8e-05

Supplementary Table 3. Human Biotinylated Bait Panel

Bait	Supplier	Supplier Code
C5A	Almac	CB-90
CCL1	Almac	CB-07
CCL11	Almac	CB-03
CCL15	Almac	CB-19
CCL17	Almac	CB-16
CCL18	Almac	CB-21
CCL19	Almac	CB-06
CCL2	Almac	CB-02
CCL20	Almac	CB-05
CCL22	Almac	CB-04
CCL25	Almac	CB-15
CCL28	Almac	CB-20
CCL3	Almac	CB-01
CCL4	Almac	CB-23
CCL5	Almac	CB-08
CCL8	Almac	CB-18
CX3CL1	Almac	CB-14
CXCL1	Protein Foundry	PFP029TBL
CXCL10	Almac	CB-10
CXCL11	Almac	CB-13
CXCL12	Almac	CB-11
CXCL12B	Almac	CB-22
CXCL13	Almac	CB-12
CXCL14	Almac	CB-17
CXCL5	Protein Foundry	PFP004TBL
CXCL8	Almac	CB-09

Supplementary Table 4. Chemokine Suppliers for Cell Migration Experiments

Chemokine	Supplier	Supplier Code
CCL1	Peprotech	300-37
CCL11	Peprotech	300-21
CCL13	Peprotech	300-24
CCL14	Peprotech	300-38B
CCL15	R&D	628-LK
CCL16	Peprotech	300-44
CCL17	Peprotech	300-30
CCL18	Peprotech	300-34
CCL19	Peprotech	300-29B
CCL2	Peprotech	300-04
CCL20	Peprotech	300-29A
CCL21	Peprotech	300-35A
CCL22	Peprotech	300-36A
CCL23	Biolegend	587002
CCL24	Peprotech	300-33
CCL25	Peprotech	300-45
CCL26	Peprotech	300-48
CCL27	Peprotech	300-54
CCL28	Peprotech	300-57
CCL3	Peprotech	300-08
CCL4	Peprotech	300-09
CCL4L1	Peprotech	300-58
CCL5	Peprotech	300-06
CCL7	Peprotech	300-17
CCL8	Peprotech	300-15
CX3CL1	Peprotech	300-31
CXCL1	Peprotech	300-11
CXCL10	Peprotech	300-12
CXCL11	Peprotech	300-46
CXCL12	Peprotech	300-28A
CXCL13	Peprotech	300-47
CXCL14	Peprotech	300-50
CXCL16	Peprotech	300-55
CXCL2	Peprotech	300-39
CXCL3	Peprotech	300-40
CXCL4	Peprotech	300-16
CXCL5	Peprotech	300-22
CXCL6	Peprotech	300-41
CXCL7	Peprotech	300-14
CXCL8	Peprotech	200-08
CXCL9	Peprotech	300-26
XCL1	Peprotech	300-20

Supplementary Table 5. Plaque Atherosclerosis Chemokine Pool Construction

Chemokine	Supplier	Code	Concentration [uM]
CCL2	Peprotech	300-04	2.6107
CCL18	Peprotech	300-34	1.85075
CXCL12	Peprotech	300-28A	1.23199
CCL3	Peprotech	300-08	0.62004
CXCL16	Peprotech	300-55	0.5649
CCL4	Peprotech	300-09	0.43035
CXCL8	Peprotech	200-08	0.34388
CCL14	Peprotech	300-38B	0.32441
CCL8	Peprotech	300-15	0.27733
CCL4L1	Peprotech	300-58	0.21912
CCL5	Peprotech	300-06	0.20515
CXCL7	Peprotech	300-14	0.18593
CCL19	Peprotech	300-29B	0.17573
CXCL10	Peprotech	300-12	0.15205
CCL13	Peprotech	300-24	0.12873
CXCL2	Peprotech	300-39	0.10247
CCL21	Peprotech	300-35A	0.08433
CXCL9	Peprotech	300-26	0.07584
CX3CL1	Peprotech	300-31	0.07273
CXCL1	Peprotech	300-11	0.06253
CXCL3	Peprotech	300-40	0.04122
CCL7	Peprotech	300-17	0.03498
CCL28	Peprotech	300-57	0.03433
CXCL4	Peprotech	300-16	0.03391
CXCL5	Peprotech	300-22	0.02478
CXCL11	Peprotech	300-46	0.01914
CCL23	Biolegend	587002	0.01151
XCL1	Peprotech	300-20	0.01034
CCL26	Peprotech	300-48	0.01013
CXCL6	Peprotech	300-41	0.0101
CCL16	Peprotech	300-44	0.00895
CXCL13	Peprotech	300-47	0.00856
CCL20	Peprotech	300-29A	0.00822
CCL22	Peprotech	300-36A	0.00705
CCL24	Peprotech	300-33	0.00689
CCL17	Peprotech	300-30	0.00334
CCL11	Peprotech	300-21	0.00316
CXCL14	Peprotech	300-50	0.0018
CCL15	Novus	NBP2-35043	0.00149
CCL27	Peprotech	300-54	7e-04
CCL1	Peprotech	300-37	0.00042

Supplementary Table 6. Islet Cytokine Stimulated Chemokine Pool Construction

Chemokine	Supplier	Code	Concentration [uM]
CXCL10	Peprotech	300-12	3.9878
CXCL11	Peprotech	300-46	1.84814
CXCL9	Peprotech	300-26	1.29189
CXCL8	Peprotech	200-08	0.70476
CX3CL1	Peprotech	300-31	0.66784
CXCL16	Peprotech	300-55	0.44907
CXCL1	Peprotech	300-11	0.3473
CCL2	Peprotech	300-04	0.16513
CXCL2	Peprotech	300-39	0.12669
CXCL6	Peprotech	300-41	0.11593
CXCL5	Peprotech	300-22	0.08007
CCL5	Peprotech	300-06	0.06408
CCL11	Peprotech	300-21	0.04686
CCL22	Peprotech	300-36A	0.03749
CCL20	Peprotech	300-29A	0.01826
CCL8	Peprotech	300-15	0.01771
CXCL3	Peprotech	300-40	0.01336
CCL19	Peprotech	300-29B	0.01117
CCL28	Peprotech	300-57	0.00646

Supplementary Table 7. Synovium Rheumatoid Chemokine Pool Construction

Chemokine	Supplier	Code	Concentration [uM]
CXCL12	Peprotech	300-28A	1.35936
CCL2	Peprotech	300-04	1.14653
CXCL9	Peprotech	300-26	0.9604
CCL13	Peprotech	300-24	0.84877
CXCL13	Peprotech	300-47	0.61406
CXCL16	Peprotech	300-55	0.42954
CXCL14	Peprotech	300-50	0.42714
CXCL7	Peprotech	300-14	0.41153
CCL28	Peprotech	300-57	0.35145
CXCL11	Peprotech	300-46	0.34221
CXCL10	Peprotech	300-12	0.30635
CXCL3	Peprotech	300-40	0.29331
CCL19	Peprotech	300-29B	0.21757
CCL8	Peprotech	300-15	0.20707
CXCL1	Peprotech	300-11	0.17791
CCL21	Peprotech	300-35A	0.16571
CXCL4	Peprotech	300-16	0.16231
CCL20	Peprotech	300-29A	0.15187
CXCL6	Peprotech	300-41	0.15083
CXCL8	Peprotech	200-08	0.14389
XCL1	Peprotech	300-20	0.12824
CCL11	Peprotech	300-21	0.12399
CCL7	Peprotech	300-17	0.12362
CX3CL1	Peprotech	300-31	0.12213
CCL26	Peprotech	300-48	0.10489
CCL1	Peprotech	300-37	0.09306
CCL25	Peprotech	300-45	0.09193
CCL22	Peprotech	300-36A	0.08378
CXCL5	Peprotech	300-22	0.0774
CXCL2	Peprotech	300-39	0.06321
CCL17	Peprotech	300-30	0.06015
CCL23	Biolegend	587002	0.03023
CCL16	Peprotech	300-44	0.01452
CCL24	Peprotech	300-33	0.01319
CCL15	Novus	NBP2-35043	0.00178
CCL5	Peprotech	300-06	7e-05

Supplementary Table 8. Peptide Sequences

Peptide	Sequence	Expected Monomeric Mass Da	Observed Mass Da	Water	PBS	DMSO	HCOOH
HD2	EEDDYTAYAPLTCYFT	1901.02	1901.4	Insoluble	Insoluble	Soluble	NA
HD2SCR	TLETDTFYECPDAYAY	1901.02	1902.2	Insoluble	Insoluble	Soluble	NA
HD2E1D	DEDDYTAYAPLTCYFT						
HD2E2D	EDDDYTAYAPLTCYFT						
HD2E2W	EWDDYTAYAPLTCYFT						
HD2Y5W	EEDDWTAYAPLTCYFT	1924.06	1923.6	Insoluble	Insoluble	Soluble	NA
HD2T6D	EEDDYDAYAPLTCYFT	1915	1914.6	Insoluble	Insoluble	Soluble	NA
HD2T6W	EEDDYWAYAPLTCYFT	1986.13	1986.5	Insoluble	Insoluble	Soluble	NA
HD2A7D	EEDDYTDYAPLTCYFT						
HD2Y8W	EEDDYTAWAPLTCYFT						
HD2A9W	EEDDYTAYWPLTCYFT	2016.15	2016.2	Insoluble	Insoluble	Insoluble	Soluble
HD2P10D	EEDDYTAYADLTCYFT						
HD2L11I	EEDDYTAYAPITCYFT	1901.02	1900.8	Insoluble	Insoluble	Soluble	NA
HD2T12I	EEDDYTAYAPLICYFT						
HD2T12V	EEDDYTAYAPLVCYFT						
HD2T12W	EEDDYTAYAPLWCYFT	1987.11	1987	Insoluble	Insoluble	Soluble	NA
HD2T16C	EEDDYTAYAPLTCYFC	1903.06	1902.6	Insoluble	Insoluble	Soluble	NA
HD2T16D	EEDDYTAYAPLTCYFD						
HD2CM1598	DEDDWTDYADLTCYFC						
HD2CM2132	EEDDYWDWADITCYFC						
HD2CM3085	DWDDWTAYWDLVCYFD	2112.24	2112.2	Insoluble	Insoluble	Insoluble	Soluble
HD2CM1516	DWDDYWAYAPLVCYFC						
HD2CM418	DDDDYWAYAPLWCYFD						
HD2CM307	DEDDWDDYAPITCYFT	1968.02	1967.8	Insoluble	Insoluble	Insoluble	Soluble
HD2CM539	DDDDWDDYAPITCYFD	1967.98	1967.8	Insoluble	Insoluble	Insoluble	Soluble
HD2CM462	DDDDWDAYAPITCYFD						

Peptide	Sequence	Expected Monomeric Mass Da	Observed Mass Da	Water	PBS	DMSO	HCOOH
HD2CM470	EDDDWDDYAPITCYFD						
HD2CM325	EEDDWDDYAPITCYFD						
HD2CM304	DEDDWDAYAPIWCYFT	2009.12	2008.8	Insoluble	Insoluble	Soluble	NA
HD2CM155	EEDDWDAYAPIWCYFT						
HD2CM452	DDDDWDAYAPIWCYFT	1995.09	1994.4	Insoluble	Insoluble	Soluble	NA
HD2CM320	EDDDWDAYAPIWCYFT						
HD2CM322	EDDDYDAYAPIWCYFD						
HD2CM423	DDDDYWDYAPLTCYFD						
HD2CM526	DDDDWWDYAPLTCYFD	2039.1	2039.2	Insoluble	Insoluble	Soluble	NA
HD2CM1578	EWDDWTDYAPLVCYFD						
HD2CM1400	DWDDYWDYAPLTCYFD						
HD2CM1530	EEDDWTDYWPITCYFD						

Supplementary Table 9a. Exact P values for phage-display mutagenesis analyses, comparisons to control

Residue	Selection method	Number of samples	P value
HD2	selected ALL	22	NA
E1D	selected ALL	22	1.06e-01
E2D	selected ALL	22	7.66e-01
E2W	selected ALL	22	1.00e+00
Y5W	selected ALL	22	9.14e-02
T6D	selected ALL	22	1.57e-04
T6W	selected ALL	22	1.81e-04
A7D	selected ALL	22	4.77e-02
Y8W	selected ALL	22	9.99e-01
A9W	selected ALL	22	3.78e-01
P10D	selected ALL	22	1.09e-05
L11I	selected ALL	22	9.26e-03
T12I	selected ALL	22	7.54e-01
T12V	selected ALL	22	7.62e-01
T12W	selected ALL	22	3.15e-01
T16C	selected ALL	22	4.76e-02
T16D	selected ALL	22	5.51e-01
HD2	selected CC	15	NA
E1D	selected CC	15	9.90e-02
E2D	selected CC	15	8.17e-01
E2W	selected CC	15	6.72e-01
Y5W	selected CC	15	6.29e-01
T6D	selected CC	15	1.40e-03
T6W	selected CC	15	7.76e-02
A7D	selected CC	15	3.65e-01
Y8W	selected CC	15	3.80e-01

Residue	Selection method	Number of samples	P value
A9W	selected CC	15	1.48e-04
P10D	selected CC	15	1.08e-12
L11I	selected CC	15	3.19e-02
T12I	selected CC	15	9.88e-01
T12V	selected CC	15	1.00e+00
T12W	selected CC	15	1.89e-01
T16C	selected CC	15	5.17e-06
T16D	selected CC	15	9.94e-01
HD2	selected CXnC	7	NA
E1D	selected CXnC	7	7.50e-01
E2D	selected CXnC	7	9.85e-01
E2W	selected CXnC	7	9.16e-01
Y5W	selected CXnC	7	4.19e-02
T6D	selected CXnC	7	1.36e-02
T6W	selected CXnC	7	2.76e-05
A7D	selected CXnC	7	4.37e-02
Y8W	selected CXnC	7	8.84e-01
A9W	selected CXnC	7	1.55e-01
P10D	selected CXnC	7	9.97e-01
L11I	selected CXnC	7	1.15e-01
T12I	selected CXnC	7	6.51e-01
T12V	selected CXnC	7	3.99e-01
T12W	selected CXnC	7	9.94e-01
T16C	selected CXnC	7	4.66e-01
T16D	selected CXnC	7	2.42e-01

Supplementary Table 9b. Exact P values for cell migration experiments, comparisons to control

ID	Experiment components	Control	Number of biological replicates	P value
Single	HD2	HD2	36	NA
Single	HD2SCR	HD2	36	2.42e-02
Single	HD2Y5W	HD2	31	1.85e-03
Single	HD2T6W	HD2	31	1.79e-03
Single	HD2T6D	HD2	9	1.00e+00
Single	HD2A9W	HD2	31	4.44e-03
Single	HD2L11I	HD2	9	2.77e-01
Single	HD2T16C	HD2	30	5.05e-05

Supplementary Table 9c. Exact P values for cell migration experiments, comparisons to control

ID	Experiment components	Control	Number of biological replicates	P value
Combinatorial	HD2Y5W	HD2Y5W	40	NA
Combinatorial	HD2	HD2Y5W	40	3.40e-01
Combinatorial	HD2SCR	HD2Y5W	40	3.84e-06
Combinatorial	HD2T6D	HD2Y5W	31	1.00e+00
Combinatorial	HD2T6W	HD2Y5W	31	1.00e+00
Combinatorial	HD2A9W	HD2Y5W	13	6.66e-01
Combinatorial	HD2L11I	HD2Y5W	31	1.85e-01
Combinatorial	HD2T12W	HD2Y5W	31	7.67e-01
Combinatorial	HD2T16C	HD2Y5W	28	1.27e-01
Combinatorial	HD2CM304	HD2Y5W	31	8.33e-01
Combinatorial	HD2CM307	HD2Y5W	31	1.10e-02
Combinatorial	HD2CM452	HD2Y5W	31	1.00e+00
Combinatorial	HD2CM526	HD2Y5W	25	8.23e-01
Combinatorial	HD2CM539	HD2Y5W	28	2.86e-01

Supplementary Table 9d. Exact P values for cell migration experiments, comparisons to control

ID	Experiment components	Control	Number of biological replicates	P value
ATHERO	base	HD2SCR	12	9.55e-14
ATHERO	ATHERO	HD2SCR	12	1.00e+00
ATHERO	HD2SCR	HD2SCR	12	NA
ATHERO	HD2	HD2SCR	12	1.04e-02
ATHERO	HD2Y5W	HD2SCR	12	8.04e-03
ATHERO	HD2T6D	HD2SCR	12	6.90e-01
ATHERO	HD2T6W	HD2SCR	12	9.78e-02
ATHERO	HD2A9W	HD2SCR	12	6.29e-01
ATHERO	HD2L11I	HD2SCR	12	2.46e-01
ATHERO	HD2T12W	HD2SCR	12	8.54e-01
ATHERO	HD2T16C	HD2SCR	12	4.25e-02
ATHERO	HD2CM304	HD2SCR	12	3.32e-05
ATHERO	HD2CM307	HD2SCR	12	4.04e-04
ATHERO	HD2CM452	HD2SCR	12	4.00e-03
ATHERO	HD2CM526	HD2SCR	12	1.70e-02
ATHERO	HD2CM539	HD2SCR	12	8.94e-04
ATHERO	EVA4	HD2SCR	12	9.58e-08
INS	base	HD2SCR	12	8.27e-10
INS	INS	HD2SCR	12	9.96e-01
INS	HD2SCR	HD2SCR	12	NA
INS	HD2	HD2SCR	12	1.00e+00
INS	HD2Y5W	HD2SCR	12	5.26e-03
INS	HD2T6D	HD2SCR	12	4.39e-01
INS	HD2T6W	HD2SCR	12	1.72e-02
INS	HD2L11I	HD2SCR	12	4.43e-01

ID	Experiment components	Control	Number of biological replicates	P value
INS	HD2T12W	HD2SCR	12	9.52e-01
INS	HD2T16C	HD2SCR	12	9.90e-01
INS	HD2CM304	HD2SCR	12	7.61e-04
INS	HD2CM307	HD2SCR	12	1.22e-03
INS	HD2CM452	HD2SCR	12	1.30e-02
INS	HD2CM526	HD2SCR	12	6.42e-01
INS	HD2CM539	HD2SCR	12	1.49e-02
INS	EVA4	HD2SCR	12	8.65e-02
RA	base	HD2SCR	9	1.36e-07
RA	RA	HD2SCR	12	1.00e+00
RA	HD2SCR	HD2SCR	12	NA
RA	HD2	HD2SCR	12	9.81e-01
RA	HD2Y5W	HD2SCR	12	7.67e-02
RA	HD2T6D	HD2SCR	12	3.85e-01
RA	HD2T6W	HD2SCR	12	9.97e-01
RA	HD2L11I	HD2SCR	12	6.21e-01
RA	HD2T12W	HD2SCR	12	5.69e-01
RA	HD2T16C	HD2SCR	9	2.62e-03
RA	HD2CM304	HD2SCR	12	1.71e-05
RA	HD2CM307	HD2SCR	12	1.75e-02
RA	HD2CM452	HD2SCR	12	2.01e-03
RA	HD2CM526	HD2SCR	12	1.00e+00
RA	HD2CM539	HD2SCR	12	6.17e-01
RA	EVA4	HD2SCR	12	7.91e-03

Supplementary Table 9e. Exact P values for pIC50 comparisons to control

Experiment components	Control	Number of biological replicates	P value
ATC,RA,HD2	HD2	3	NA
ATC,RA,HD2CM304	HD2	3	3.06e-03
ATC,RA,HD2CM307	HD2	3	4.60e-01
THP1,INS,HD2	HD2	3	NA
THP1,INS,HD2CM304	HD2	3	2.41e-01
THP1,INS,HD2CM307	HD2	3	6.80e-02
THP1,RA,HD2	HD2	3	NA
THP1,RA,HD2CM304	HD2	3	5.15e-01
THP1,RA,HD2CM307	HD2	3	1.72e-01
THP1,ATHERO,HD2	HD2	3	NA
THP1,ATHERO,HD2CM304	HD2	3	8.91e-01
THP1,ATHERO,HD2CM307	HD2	3	2.19e-02
ATC,INS,HD2	HD2	3	NA
ATC,INS,HD2CM304	HD2	3	3.15e-02
ATC,INS,HD2CM307	HD2	3	6.11e-01
ATC,ATHERO,HD2	HD2	3	NA
ATC,ATHERO,HD2CM304	HD2	3	3.86e-02
ATC,ATHERO,HD2CM307	HD2	3	5.28e-01

Supplementary Table 9f. Exact P values for Arpeggio bond numbers, comparisons to control

Test	Control	Number of data points	P value	Bond type	Amino acid type
HD2CM304	HD2	46	1.74e-03	all	all
HD2CM307	HD2	46	5.95e-03	all	all
HD2CM304	HD2	46	2.13e-02	hydrophobic	R3
HD2CM307	HD2	46	8.59e-03	hydrophobic	R3
HD2CM304	HD2	46	4.02e-04	hydrophobic	R5
HD2CM307	HD2	46	8.90e-05	hydrophobic	R5
HD2CM304	HD2	46	2.81e-02	hydrophobic	R6
HD2CM304	HD2	46	3.96e-08	hydrophobic	R12
HD2CM307	HD2	46	1.12e-02	hydrophobic	R15
HD2CM304	HD2	46	1.22e-04	hydrophobic	all
HD2CM307	HD2	46	7.07e-03	hydrophobic	all

Supplementary Table 9g. Exact P values for cell migration experiments, comparisons to control

ID	Experiment components	Control	Number of biological replicates	P value
R13	THP1	HD2	4	0.00e+00
R13	THP1,CCL14	HD2	4	9.98e-01
R13	THP1,CCL14,HD2	HD2	4	NA
R13	THP1,CCL14,HD2SCR	HD2	4	1.00e+00
R13	THP1,CCL14,HD2Y5W	HD2	3	9.79e-01
R13	THP1,CCL14,HD2T6W	HD2	3	1.00e+00
R13	THP1,CCL14,HD2A9W	HD2	3	1.00e+00
R13	THP1,CCL14,HD2T16C	HD2	3	2.76e-03
R15	THP1	HD2	3	1.00e-06
R15	THP1,CCL23	HD2	3	1.00e+00
R15	THP1,CCL23,HD2	HD2	3	NA
R15	THP1,CCL23,HD2SCR	HD2	3	8.55e-01
R15	THP1,CCL23,HD2Y5W	HD2	3	1.47e-01
R15	THP1,CCL23,HD2T6W	HD2	3	5.87e-01
R15	THP1,CCL23,HD2A9W	HD2	3	1.55e-02
R15	THP1,CCL23,HD2T16C	HD2	3	2.55e-02
R16	J:CXCR1	HD2	3	2.50e-05
R16	J:CXCR1,CXCL6	HD2	3	1.00e-05
R16	J:CXCR1,CXCL6,HD2	HD2	3	NA
R16	J:CXCR1,CXCL6,HD2SCR	HD2	3	2.12e-02
R16	J:CXCR1,CXCL6,HD2Y5W	HD2	3	2.06e-01
R16	J:CXCR1,CXCL6,HD2T6W	HD2	3	2.36e-04
R16	J:CXCR1,CXCL6,HD2T6D	HD2	3	1.00e+00
R16	J:CXCR1,CXCL6,HD2A9W	HD2	3	2.47e-04
R16	J:CXCR1,CXCL6,HD2L11I	HD2	3	1.00e+00
R16	J:CXCR1,CXCL6,HD2T16C	HD2	3	9.20e-05
R20	ATC	HD2	5	9.79e-03
R20	ATC,CCL19	HD2	5	4.85e-02
R20	ATC,CCL19,HD2	HD2	5	NA
R20	ATC,CCL19,HD2SCR	HD2	5	9.99e-01
R20	ATC,CCL19,HD2Y5W	HD2	3	1.78e-02
R20	ATC,CCL19,HD2T6W	HD2	3	1.55e-02
R20	ATC,CCL19,HD2A9W	HD2	3	2.16e-02
R20	ATC,CCL19,HD2T16C	HD2	3	1.31e-02
R21	ATC	HD2	4	4.19e-03
R21	ATC,CXCL11	HD2	4	8.92e-02
R21	ATC,CXCL11,HD2	HD2	4	NA
R21	ATC,CXCL11,HD2SCR	HD2	4	1.49e-01
R21	ATC,CXCL11,HD2Y5W	HD2	4	1.18e-02
R21	ATC,CXCL11,HD2T6W	HD2	4	6.63e-04
R21	ATC,CXCL11,HD2A9W	HD2	4	5.73e-04
R21	ATC,CXCL11,HD2T16C	HD2	3	1.45e-03
R25	THP1	HD2	3	2.37e-01
R25	THP1,CCL5	HD2	3	0.00e+00
R25	THP1,CCL5,HD2	HD2	3	NA

ID	Experiment components	Control	Number of biological replicates	P value
R25	THP1,CCL5,HD2SCR	HD2	3	0.00e+00
R25	THP1,CCL5,HD2Y5W	HD2	3	1.32e-01
R25	THP1,CCL5,HD2T6W	HD2	3	1.00e+00
R25	THP1,CCL5,HD2A9W	HD2	3	9.95e-01
R25	THP1,CCL5,HD2T16C	HD2	3	1.00e-05
R26	THP1	HD2	3	1.45e-02
R26	THP1,CCL7	HD2	3	4.03e-04
R26	THP1,CCL7,HD2	HD2	3	NA
R26	THP1,CCL7,HD2SCR	HD2	3	3.22e-04
R26	THP1,CCL7,HD2Y5W	HD2	3	6.66e-01
R26	THP1,CCL7,HD2T6W	HD2	3	8.87e-01
R26	THP1,CCL7,HD2A9W	HD2	3	7.33e-01
R26	THP1,CCL7,HD2T16C	HD2	3	1.00e+00
R27	THP1	HD2	3	1.00e+00
R27	THP1,CCL8	HD2	3	0.00e+00
R27	THP1,CCL8,HD2	HD2	3	NA
R27	THP1,CCL8,HD2SCR	HD2	3	0.00e+00
R27	THP1,CCL8,HD2Y5W	HD2	3	8.86e-01
R27	THP1,CCL8,HD2T6W	HD2	3	9.45e-01
R27	THP1,CCL8,HD2A9W	HD2	3	9.12e-01
R27	THP1,CCL8,HD2T16C	HD2	3	9.61e-01
R44	ATC	HD2	5	0.00e+00
R44	ATC,CXCL9	HD2	5	3.86e-02
R44	ATC,CXCL9,HD2	HD2	5	NA
R44	ATC,CXCL9,HD2SCR	HD2	5	9.24e-01
R44	ATC,CXCL9,HD2Y5W	HD2	3	0.00e+00
R44	ATC,CXCL9,HD2T6W	HD2	3	9.00e-06
R44	ATC,CXCL9,HD2T6D	HD2	3	1.00e+00
R44	ATC,CXCL9,HD2A9W	HD2	3	4.79e-02
R44	ATC,CXCL9,HD2L11I	HD2	3	4.00e-06
R44	ATC,CXCL9,HD2T12W	HD2	3	4.14e-03
R44	ATC,CXCL9,HD2T16C	HD2	3	0.00e+00
R45	ATC	HD2	3	3.00e-06
R45	ATC,CCL21	HD2	3	9.61e-01
R45	ATC,CCL21,HD2	HD2	3	NA
R45	ATC,CCL21,HD2SCR	HD2	3	1.00e+00
R45	ATC,CCL21,HD2Y5W	HD2	3	5.40e-05
R45	ATC,CCL21,HD2T6W	HD2	3	8.87e-02
R45	ATC,CCL21,HD2T6D	HD2	3	9.96e-01
R45	ATC,CCL21,HD2A9W	HD2	3	1.07e-01
R45	ATC,CCL21,HD2L11I	HD2	3	4.29e-01
R45	ATC,CCL21,HD2T12W	HD2	3	8.36e-01
R45	ATC,CCL21,HD2T16C	HD2	3	4.00e-06
R47	ATC	HD2Y5W	6	0.00e+00
R47	ATC,CXCL12	HD2Y5W	6	0.00e+00
R47	ATC,CXCL12,HD2Y5W	HD2Y5W	6	NA
R47	ATC,CXCL12,HD2SCR	HD2Y5W	6	2.00e-06
R47	ATC,CXCL12,HD2	HD2Y5W	6	7.00e-06
R47	ATC,CXCL12,HD2T6W	HD2Y5W	3	9.99e-01
R47	ATC,CXCL12,HD2T6D	HD2Y5W	3	2.17e-01
R47	ATC,CXCL12,HD2L11I	HD2Y5W	3	1.54e-02
R47	ATC,CXCL12,HD2T12W	HD2Y5W	3	9.95e-01

ID	Experiment components	Control	Number of biological replicates	P value
R47	ATC,CXCL12,HD2T16C	HD2Y5W	3	1.48e-04
R47	ATC,CXCL12,HD2CM304	HD2Y5W	3	2.34e-04
R47	ATC,CXCL12,HD2CM307	HD2Y5W	3	9.99e-01
R47	ATC,CXCL12,HD2CM452	HD2Y5W	3	9.12e-04
R47	ATC,CXCL12,HD2CM526	HD2Y5W	3	1.00e+00
R47	ATC,CXCL12,HD2CM539	HD2Y5W	3	2.15e-01
R61	THP1	HD2Y5W	3	0.00e+00
R61	THP1,CCL2	HD2Y5W	3	7.77e-01
R61	THP1,CCL2,HD2Y5W	HD2Y5W	3	NA
R61	THP1,CCL2,HD2SCR	HD2Y5W	3	4.60e-01
R61	THP1,CCL2,HD2	HD2Y5W	3	1.00e+00
R61	THP1,CCL2,HD2T6W	HD2Y5W	3	1.00e+00
R61	THP1,CCL2,HD2T6D	HD2Y5W	3	1.00e+00
R61	THP1,CCL2,HD2A9W	HD2Y5W	3	1.00e+00
R61	THP1,CCL2,HD2L11I	HD2Y5W	3	1.00e+00
R61	THP1,CCL2,HD2T12W	HD2Y5W	3	9.98e-01
R61	THP1,CCL2,HD2T16C	HD2Y5W	3	7.79e-01
R61	THP1,CCL2,HD2CM304	HD2Y5W	3	1.00e+00
R61	THP1,CCL2,HD2CM307	HD2Y5W	3	1.11e-01
R61	THP1,CCL2,HD2CM452	HD2Y5W	3	9.79e-01
R61	THP1,CCL2,HD2CM526	HD2Y5W	3	1.00e+00
R61	THP1,CCL2,HD2CM539	HD2Y5W	3	7.82e-01
R62	ATC	HD2Y5W	3	4.00e-06
R62	ATC,CXCL11	HD2Y5W	3	3.50e-01
R62	ATC,CXCL11,HD2Y5W	HD2Y5W	3	NA
R62	ATC,CXCL11,HD2SCR	HD2Y5W	3	9.28e-01
R62	ATC,CXCL11,HD2	HD2Y5W	3	4.47e-01
R62	ATC,CXCL11,HD2T6W	HD2Y5W	3	8.23e-01
R62	ATC,CXCL11,HD2T6D	HD2Y5W	3	1.00e+00
R62	ATC,CXCL11,HD2L11I	HD2Y5W	3	1.00e+00
R62	ATC,CXCL11,HD2T12W	HD2Y5W	3	9.97e-01
R62	ATC,CXCL11,HD2T16C	HD2Y5W	3	3.72e-02
R62	ATC,CXCL11,HD2CM304	HD2Y5W	3	2.17e-03
R62	ATC,CXCL11,HD2CM452	HD2Y5W	3	3.99e-02
R62	ATC,CXCL11,HD2CM539	HD2Y5W	3	1.46e-04
R64	ATC	HD2Y5W	3	1.00e+00
R64	ATC,CCL21	HD2Y5W	3	3.00e-06
R64	ATC,CCL21,HD2Y5W	HD2Y5W	3	NA
R64	ATC,CCL21,HD2SCR	HD2Y5W	3	9.00e-05
R64	ATC,CCL21,HD2	HD2Y5W	3	8.50e-05
R64	ATC,CCL21,HD2T6W	HD2Y5W	3	5.21e-01
R64	ATC,CCL21,HD2T6D	HD2Y5W	3	2.98e-04
R64	ATC,CCL21,HD2L11I	HD2Y5W	3	2.08e-02
R64	ATC,CCL21,HD2T12W	HD2Y5W	3	2.87e-03
R64	ATC,CCL21,HD2CM304	HD2Y5W	3	9.99e-01
R64	ATC,CCL21,HD2CM452	HD2Y5W	3	9.87e-01
R66	THP1	HD2Y5W	4	1.29e-02
R66	THP1,CCL3	HD2Y5W	4	1.00e+00
R66	THP1,CCL3,HD2Y5W	HD2Y5W	4	NA
R66	THP1,CCL3,HD2SCR	HD2Y5W	4	9.96e-01
R66	THP1,CCL3,HD2	HD2Y5W	4	4.17e-01
R66	THP1,CCL3,HD2T6W	HD2Y5W	4	8.04e-01

ID	Experiment components	Control	Number of biological replicates	P value
R66	THP1,CCL3,HD2T6D	HD2Y5W	4	4.20e-01
R66	THP1,CCL3,HD2A9W	HD2Y5W	4	9.99e-01
R66	THP1,CCL3,HD2L11I	HD2Y5W	4	9.94e-01
R66	THP1,CCL3,HD2T12W	HD2Y5W	4	1.43e-02
R66	THP1,CCL3,HD2T16C	HD2Y5W	4	4.02e-01
R66	THP1,CCL3,HD2CM304	HD2Y5W	4	8.46e-02
R66	THP1,CCL3,HD2CM307	HD2Y5W	4	7.38e-02
R66	THP1,CCL3,HD2CM452	HD2Y5W	4	9.96e-01
R66	THP1,CCL3,HD2CM526	HD2Y5W	4	9.99e-01
R66	THP1,CCL3,HD2CM539	HD2Y5W	4	8.24e-01
R67	ATC	HD2Y5W	3	0.00e+00
R67	ATC,CXCL10	HD2Y5W	3	1.00e+00
R67	ATC,CXCL10,HD2Y5W	HD2Y5W	3	NA
R67	ATC,CXCL10,HD2SCR	HD2Y5W	3	1.00e+00
R67	ATC,CXCL10,HD2	HD2Y5W	3	1.00e+00
R67	ATC,CXCL10,HD2T6W	HD2Y5W	3	9.07e-01
R67	ATC,CXCL10,HD2T6D	HD2Y5W	3	9.74e-01
R67	ATC,CXCL10,HD2A9W	HD2Y5W	3	5.02e-02
R67	ATC,CXCL10,HD2L11I	HD2Y5W	3	1.00e+00
R67	ATC,CXCL10,HD2T12W	HD2Y5W	3	1.00e+00
R67	ATC,CXCL10,HD2T16C	HD2Y5W	3	1.00e+00
R67	ATC,CXCL10,HD2CM304	HD2Y5W	3	1.00e+00
R67	ATC,CXCL10,HD2CM307	HD2Y5W	3	4.38e-03
R67	ATC,CXCL10,HD2CM452	HD2Y5W	3	5.05e-01
R67	ATC,CXCL10,HD2CM526	HD2Y5W	3	4.11e-03
R67	ATC,CXCL10,HD2CM539	HD2Y5W	3	7.72e-03
R79	THP1	HD2Y5W	3	4.09e-04
R79	THP1,CCL7	HD2Y5W	3	1.07e-04
R79	THP1,CCL7,HD2Y5W	HD2Y5W	3	NA
R79	THP1,CCL7,HD2SCR	HD2Y5W	3	1.33e-03
R79	THP1,CCL7,HD2	HD2Y5W	3	9.67e-01
R79	THP1,CCL7,HD2T6W	HD2Y5W	3	9.95e-01
R79	THP1,CCL7,HD2T6D	HD2Y5W	3	9.96e-01
R79	THP1,CCL7,HD2L11I	HD2Y5W	3	4.56e-01
R79	THP1,CCL7,HD2T12W	HD2Y5W	3	4.55e-01
R79	THP1,CCL7,HD2T16C	HD2Y5W	3	7.22e-01
R79	THP1,CCL7,HD2CM304	HD2Y5W	3	4.48e-01
R79	THP1,CCL7,HD2CM307	HD2Y5W	3	2.03e-03
R79	THP1,CCL7,HD2CM452	HD2Y5W	3	1.00e+00
R79	THP1,CCL7,HD2CM526	HD2Y5W	3	1.00e+00
R79	THP1,CCL7,HD2CM539	HD2Y5W	3	2.05e-02
R86	THP1	HD2Y5W	3	2.71e-01
R86	THP1,CCL8	HD2Y5W	3	1.98e-03
R86	THP1,CCL8,HD2Y5W	HD2Y5W	3	NA
R86	THP1,CCL8,HD2SCR	HD2Y5W	3	3.39e-03
R86	THP1,CCL8,HD2	HD2Y5W	3	2.71e-02
R86	THP1,CCL8,HD2T6W	HD2Y5W	3	9.27e-01
R86	THP1,CCL8,HD2T6D	HD2Y5W	3	8.48e-01
R86	THP1,CCL8,HD2L11I	HD2Y5W	3	1.48e-01
R86	THP1,CCL8,HD2T12W	HD2Y5W	3	1.83e-01
R86	THP1,CCL8,HD2T16C	HD2Y5W	3	3.21e-03
R86	THP1,CCL8,HD2CM304	HD2Y5W	3	6.17e-02

ID	Experiment components	Control	Number of biological replicates	P value
R86	THP1,CCL8,HD2CM307	HD2Y5W	3	1.00e+00
R86	THP1,CCL8,HD2CM452	HD2Y5W	3	1.32e-01
R86	THP1,CCL8,HD2CM526	HD2Y5W	3	6.76e-01
R86	THP1,CCL8,HD2CM539	HD2Y5W	3	9.17e-01
R94	THP1	HD2	3	4.03e-01
R94	THP1,CCL8	HD2	3	6.24e-04
R94	THP1,CCL8,HD2	HD2	3	NA
R94	THP1,CCL8,HD2SCR	HD2	3	5.70e-05
R94	THP1,CCL8,HD2Y5W	HD2	3	1.22e-02
R94	THP1,CCL8,HD2CM307	HD2	3	6.04e-01
R94	THP1,CCL8,HD2CM3085	HD2	3	3.77e-04
R95	THP1	HD2	3	5.38e-03
R95	THP1,CCL7	HD2	3	2.17e-04
R95	THP1,CCL7,HD2	HD2	3	NA
R95	THP1,CCL7,HD2SCR	HD2	3	6.96e-04
R95	THP1,CCL7,HD2Y5W	HD2	3	5.15e-01
R95	THP1,CCL7,HD2CM307	HD2	3	1.12e-02
R95	THP1,CCL7,HD2CM3085	HD2	3	7.88e-01
R97	ATC	HD2SCR	3	0.00e+00
R97	ATC,INS	HD2SCR	3	2.77e-01
R97	ATC,INS,HD2SCR	HD2SCR	3	NA
R97	ATC,INS,HD2	HD2SCR	3	0.00e+00
R97	ATC,INS,HD2Y5W	HD2SCR	3	0.00e+00
R97	ATC,INS,HD2T6W	HD2SCR	3	0.00e+00
R97	ATC,INS,HD2T6D	HD2SCR	3	3.50e-05
R97	ATC,INS,HD2L11I	HD2SCR	3	0.00e+00
R97	ATC,INS,HD2T12W	HD2SCR	3	8.00e-06
R97	ATC,INS,HD2T16C	HD2SCR	3	0.00e+00
R97	ATC,INS,HD2CM304	HD2SCR	3	0.00e+00
R97	ATC,INS,HD2CM307	HD2SCR	3	0.00e+00
R97	ATC,INS,HD2CM452	HD2SCR	3	0.00e+00
R97	ATC,INS,HD2CM526	HD2SCR	3	1.50e-05
R97	ATC,INS,HD2CM539	HD2SCR	3	0.00e+00
R97	ATC,INS,EVA4	HD2SCR	3	1.00e-06
R105	Granulocytes	HD2SCR	3	2.40e-04
R105	Granulocytes,RA	HD2SCR	3	6.59e-01
R105	Granulocytes,RA,HD2SCR	HD2SCR	3	NA
R105	Granulocytes,RA,HD2	HD2SCR	3	9.78e-01
R105	Granulocytes,RA,HD2Y5W	HD2SCR	3	9.51e-02
R105	Granulocytes,RA,HD2T6W	HD2SCR	3	5.41e-01
R105	Granulocytes,RA,HD2T6D	HD2SCR	3	9.80e-05
R105	Granulocytes,RA,HD2L11I	HD2SCR	3	1.00e+00
R105	Granulocytes,RA,HD2T12W	HD2SCR	3	7.67e-01
R105	Granulocytes,RA,HD2T16C	HD2SCR	3	1.24e-01
R105	Granulocytes,RA,HD2CM304	HD2SCR	3	1.27e-02
R105	Granulocytes,RA,HD2CM307	HD2SCR	3	8.96e-01
R105	Granulocytes,RA,HD2CM452	HD2SCR	3	1.00e+00
R105	Granulocytes,RA,HD2CM526	HD2SCR	3	1.00e+00

ID	Experiment components	Control	Number of biological replicates	P value
R105	Granulocytes,RA,HD2CM539	HD2SCR	3	9.99e-01
R105	Granulocytes,RA,EVA4	HD2SCR	3	5.17e-01
R106	Monocytes	HD2SCR	3	5.45e-03
R106	Monocytes,RA	HD2SCR	3	1.00e+00
R106	Monocytes,RA,HD2SCR	HD2SCR	3	NA
R106	Monocytes,RA,HD2	HD2SCR	3	1.00e+00
R106	Monocytes,RA,HD2Y5W	HD2SCR	3	9.98e-01
R106	Monocytes,RA,HD2T6W	HD2SCR	3	1.00e+00
R106	Monocytes,RA,HD2T6D	HD2SCR	3	9.95e-01
R106	Monocytes,RA,HD2L11I	HD2SCR	3	8.34e-01
R106	Monocytes,RA,HD2T12W	HD2SCR	3	7.60e-01
R106	Monocytes,RA,HD2T16C	HD2SCR	3	5.74e-01
R106	Monocytes,RA,HD2CM304	HD2SCR	3	5.31e-01
R106	Monocytes,RA,HD2CM307	HD2SCR	3	3.85e-01
R106	Monocytes,RA,HD2CM452	HD2SCR	3	9.23e-01
R106	Monocytes,RA,HD2CM526	HD2SCR	3	1.00e+00
R106	Monocytes,RA,HD2CM539	HD2SCR	3	6.07e-01
R106	Monocytes,RA,EVA4	HD2SCR	3	7.62e-01
R107	Lymphocytes	HD2SCR	3	1.60e-05
R107	Lymphocytes,RA	HD2SCR	3	2.88e-01
R107	Lymphocytes,RA,HD2SCR	HD2SCR	3	NA
R107	Lymphocytes,RA,HD2	HD2SCR	3	9.95e-01
R107	Lymphocytes,RA,HD2Y5W	HD2SCR	3	1.00e+00
R107	Lymphocytes,RA,HD2T6W	HD2SCR	3	5.49e-01
R107	Lymphocytes,RA,HD2T6D	HD2SCR	3	9.81e-01
R107	Lymphocytes,RA,HD2L11I	HD2SCR	3	7.03e-01
R107	Lymphocytes,RA,HD2T12W	HD2SCR	3	5.34e-01
R107	Lymphocytes,RA,HD2T16C	HD2SCR	3	7.39e-02
R107	Lymphocytes,RA,HD2CM304	HD2SCR	3	2.09e-03
R107	Lymphocytes,RA,HD2CM307	HD2SCR	3	1.04e-01
R107	Lymphocytes,RA,HD2CM452	HD2SCR	3	8.24e-03
R107	Lymphocytes,RA,HD2CM526	HD2SCR	3	1.00e+00
R107	Lymphocytes,RA,HD2CM539	HD2SCR	3	1.00e+00
R107	Lymphocytes,RA,EVA4	HD2SCR	3	6.26e-01
R108	Monocytes	HD2SCR	3	4.00e-06
R108	Monocytes,ATHERO	HD2SCR	3	9.94e-01
R108	Monocytes,ATHERO,HD2SCR	HD2SCR	3	NA
R108	Monocytes,ATHERO,HD2	HD2SCR	3	9.99e-01
R108	Monocytes,ATHERO,HD2Y5W	HD2SCR	3	6.44e-01
R108	Monocytes,ATHERO,HD2T6W	HD2SCR	3	1.00e+00
R108	Monocytes,ATHERO,HD2T6D	HD2SCR	3	1.00e+00

ID	Experiment components	Control	Number of biological replicates	P value
R108	Monocytes,ATHERO,HD2A9W	HD2SCR	3	9.99e-01
R108	Monocytes,ATHERO,HD2L11I	HD2SCR	3	1.00e+00
R108	Monocytes,ATHERO,HD2T12W	HD2SCR	3	6.75e-01
R108	Monocytes,ATHERO,HD2T16C	HD2SCR	3	9.91e-01
R108	Monocytes,ATHERO,HD2CM304	HD2SCR	3	4.05e-01
R108	Monocytes,ATHERO,HD2CM307	HD2SCR	3	9.98e-01
R108	Monocytes,ATHERO,HD2CM452	HD2SCR	3	3.98e-01
R108	Monocytes,ATHERO,HD2CM526	HD2SCR	3	6.84e-01
R108	Monocytes,ATHERO,HD2CM539	HD2SCR	3	3.46e-01
R108	Monocytes,ATHERO,EVA4	HD2SCR	3	2.99e-03
R109	Granulocytes	HD2SCR	3	3.53e-04
R109	Granulocytes,ATHERO	HD2SCR	3	1.00e+00
R109	Granulocytes,ATHERO,HD2SCR	HD2SCR	3	NA
R109	Granulocytes,ATHERO,HD2	HD2SCR	3	9.45e-01
R109	Granulocytes,ATHERO,HD2Y5W	HD2SCR	3	1.33e-01
R109	Granulocytes,ATHERO,HD2T6W	HD2SCR	3	9.79e-01
R109	Granulocytes,ATHERO,HD2T6D	HD2SCR	3	1.00e+00
R109	Granulocytes,ATHERO,HD2A9W	HD2SCR	3	9.79e-02
R109	Granulocytes,ATHERO,HD2L11I	HD2SCR	3	9.99e-01
R109	Granulocytes,ATHERO,HD2T12W	HD2SCR	3	1.00e+00
R109	Granulocytes,ATHERO,HD2T16C	HD2SCR	3	9.71e-01
R109	Granulocytes,ATHERO,HD2CM304	HD2SCR	3	6.72e-01
R109	Granulocytes,ATHERO,HD2CM307	HD2SCR	3	2.60e-01
R109	Granulocytes,ATHERO,HD2CM452	HD2SCR	3	1.00e+00
R109	Granulocytes,ATHERO,HD2CM526	HD2SCR	3	1.46e-01
R109	Granulocytes,ATHERO,HD2CM539	HD2SCR	3	3.91e-01
R109	Granulocytes,ATHERO,EVA4	HD2SCR	3	9.08e-02
R110	Lymphocytes	HD2SCR	3	6.00e-06
R110	Lymphocytes,ATHERO	HD2SCR	3	1.00e+00

ID	Experiment components	Control	Number of biological replicates	P value
R110	Lymphocytes,ATHERO,HD2 SCR	HD2SCR	3	NA
R110	Lymphocytes,ATHERO,HD2	HD2SCR	3	3.59e-02
R110	Lymphocytes,ATHERO,HD2 Y5W	HD2SCR	3	1.24e-01
R110	Lymphocytes,ATHERO,HD2 T6W	HD2SCR	3	3.24e-01
R110	Lymphocytes,ATHERO,HD2 T6D	HD2SCR	3	9.99e-01
R110	Lymphocytes,ATHERO,HD2 A9W	HD2SCR	3	5.49e-02
R110	Lymphocytes,ATHERO,HD2 L11I	HD2SCR	3	1.34e-01
R110	Lymphocytes,ATHERO,HD2 T12W	HD2SCR	3	9.03e-01
R110	Lymphocytes,ATHERO,HD2 T16C	HD2SCR	3	1.26e-02
R110	Lymphocytes,ATHERO,HD2 CM304	HD2SCR	3	1.57e-03
R110	Lymphocytes,ATHERO,HD2 CM307	HD2SCR	3	5.70e-02
R110	Lymphocytes,ATHERO,HD2 CM452	HD2SCR	3	2.75e-03
R110	Lymphocytes,ATHERO,HD2 CM526	HD2SCR	3	7.16e-01
R110	Lymphocytes,ATHERO,HD2 CM539	HD2SCR	3	3.69e-01
R110	Lymphocytes,ATHERO,EVA 4	HD2SCR	3	1.51e-04
R124	ATC	HD2SCR	3	0.00e+00
R124	ATC,ATHERO	HD2SCR	3	1.00e+00
R124	ATC,ATHERO,HD2SCR	HD2SCR	3	NA
R124	ATC,ATHERO,HD2	HD2SCR	3	0.00e+00
R124	ATC,ATHERO,HD2Y5W	HD2SCR	3	8.51e-04
R124	ATC,ATHERO,HD2T6W	HD2SCR	3	0.00e+00
R124	ATC,ATHERO,HD2T6D	HD2SCR	3	1.90e-05
R124	ATC,ATHERO,HD2A9W	HD2SCR	3	0.00e+00
R124	ATC,ATHERO,HD2L11I	HD2SCR	3	0.00e+00
R124	ATC,ATHERO,HD2T12W	HD2SCR	3	0.00e+00
R124	ATC,ATHERO,HD2T16C	HD2SCR	3	0.00e+00
R124	ATC,ATHERO,HD2CM304	HD2SCR	3	0.00e+00
R124	ATC,ATHERO,HD2CM307	HD2SCR	3	0.00e+00
R124	ATC,ATHERO,HD2CM452	HD2SCR	3	0.00e+00
R124	ATC,ATHERO,HD2CM526	HD2SCR	3	5.40e-05
R124	ATC,ATHERO,HD2CM539	HD2SCR	3	0.00e+00
R124	ATC,ATHERO,EVA4	HD2SCR	3	0.00e+00
R135	ATC,RA	HD2SCR	3	1.93e-04
R135	ATC,RA,HD2SCR	HD2SCR	3	NA
R135	ATC,RA,HD2	HD2SCR	3	6.92e-01
R135	ATC,RA,HD2Y5W	HD2SCR	3	0.00e+00
R135	ATC,RA,HD2T6W	HD2SCR	3	4.27e-02
R135	ATC,RA,HD2T6D	HD2SCR	3	9.44e-01

ID	Experiment components	Control	Number of biological replicates	P value
R135	ATC,RA,HD2L11I	HD2SCR	3	8.08e-02
R135	ATC,RA,HD2T12W	HD2SCR	3	3.73e-01
R135	ATC,RA,HD2CM304	HD2SCR	3	0.00e+00
R135	ATC,RA,HD2CM307	HD2SCR	3	7.00e-06
R135	ATC,RA,HD2CM452	HD2SCR	3	0.00e+00
R135	ATC,RA,HD2CM526	HD2SCR	3	7.08e-01
R135	ATC,RA,HD2CM539	HD2SCR	3	2.92e-03
R135	ATC,RA,EVA4	HD2SCR	3	0.00e+00
R137	Granulocytes	HD2SCR	3	8.60e-05
R137	Granulocytes,INS	HD2SCR	3	8.10e-01
R137	Granulocytes,INS,HD2SCR	HD2SCR	3	NA
R137	Granulocytes,INS,HD2	HD2SCR	3	1.51e-01
R137	Granulocytes,INS,HD2Y5W	HD2SCR	3	8.00e-01
R137	Granulocytes,INS,HD2T6W	HD2SCR	3	1.00e+00
R137	Granulocytes,INS,HD2T6D	HD2SCR	3	1.00e+00
R137	Granulocytes,INS,HD2L11I	HD2SCR	3	9.99e-01
R137	Granulocytes,INS,HD2T12W	HD2SCR	3	1.00e+00
R137	Granulocytes,INS,HD2T16C	HD2SCR	3	9.12e-01
R137	Granulocytes,INS,HD2CM304	HD2SCR	3	1.00e+00
R137	Granulocytes,INS,HD2CM307	HD2SCR	3	9.97e-01
R137	Granulocytes,INS,HD2CM452	HD2SCR	3	1.00e+00
R137	Granulocytes,INS,HD2CM526	HD2SCR	3	1.00e+00
R137	Granulocytes,INS,HD2CM539	HD2SCR	3	1.00e+00
R137	Granulocytes,INS,EVA4	HD2SCR	3	1.00e+00
R138	Monocytes	HD2SCR	3	2.80e-05
R138	Monocytes,INS	HD2SCR	3	8.06e-01
R138	Monocytes,INS,HD2SCR	HD2SCR	3	NA
R138	Monocytes,INS,HD2	HD2SCR	3	9.94e-01
R138	Monocytes,INS,HD2Y5W	HD2SCR	3	4.03e-02
R138	Monocytes,INS,HD2T6W	HD2SCR	3	1.48e-01
R138	Monocytes,INS,HD2T6D	HD2SCR	3	1.67e-02
R138	Monocytes,INS,HD2L11I	HD2SCR	3	6.67e-01
R138	Monocytes,INS,HD2T12W	HD2SCR	3	5.37e-01
R138	Monocytes,INS,HD2T16C	HD2SCR	3	1.00e+00
R138	Monocytes,INS,HD2CM304	HD2SCR	3	5.80e-02
R138	Monocytes,INS,HD2CM307	HD2SCR	3	1.44e-03
R138	Monocytes,INS,HD2CM452	HD2SCR	3	8.98e-02
R138	Monocytes,INS,HD2CM526	HD2SCR	3	2.04e-01
R138	Monocytes,INS,HD2CM539	HD2SCR	3	2.47e-03
R138	Monocytes,INS,EVA4	HD2SCR	3	8.10e-05
R139	Lymphocytes	HD2SCR	3	4.23e-02
R139	Lymphocytes,INS	HD2SCR	3	9.92e-01
R139	Lymphocytes,INS,HD2SCR	HD2SCR	3	NA
R139	Lymphocytes,INS,HD2	HD2SCR	3	1.00e+00
R139	Lymphocytes,INS,HD2Y5W	HD2SCR	3	1.00e+00
R139	Lymphocytes,INS,HD2T6W	HD2SCR	3	9.89e-01
R139	Lymphocytes,INS,HD2T6D	HD2SCR	3	1.00e+00

ID	Experiment components	Control	Number of biological replicates	P value
R139	Lymphocytes,INS,HD2L11I	HD2SCR	3	1.00e+00
R139	Lymphocytes,INS,HD2T12W	HD2SCR	3	9.99e-01
R139	Lymphocytes,INS,HD2T16C	HD2SCR	3	6.48e-01
R139	Lymphocytes,INS,HD2CM304	HD2SCR	3	5.78e-01
R139	Lymphocytes,INS,HD2CM307	HD2SCR	3	7.82e-01
R139	Lymphocytes,INS,HD2CM452	HD2SCR	3	9.16e-01
R139	Lymphocytes,INS,HD2CM526	HD2SCR	3	1.00e+00
R139	Lymphocytes,INS,HD2CM539	HD2SCR	3	9.84e-01
R139	Lymphocytes,INS,EVA4	HD2SCR	3	1.00e+00
R148	THP1	HD2Y5W	3	0.00e+00
R148	THP1,CCL23	HD2Y5W	3	6.87e-01
R148	THP1,CCL23,HD2Y5W	HD2Y5W	3	NA
R148	THP1,CCL23,HD2SCR	HD2Y5W	3	5.15e-01
R148	THP1,CCL23,HD2	HD2Y5W	3	8.27e-01
R148	THP1,CCL23,HD2T6W	HD2Y5W	3	8.90e-01
R148	THP1,CCL23,HD2T6D	HD2Y5W	3	1.49e-01
R148	THP1,CCL23,HD2A9W	HD2Y5W	3	1.00e+00
R148	THP1,CCL23,HD2L11I	HD2Y5W	3	4.06e-01
R148	THP1,CCL23,HD2T12W	HD2Y5W	3	8.31e-01
R148	THP1,CCL23,HD2T16C	HD2Y5W	3	1.00e+00
R148	THP1,CCL23,HD2CM304	HD2Y5W	3	3.55e-01
R148	THP1,CCL23,HD2CM307	HD2Y5W	3	5.26e-01
R148	THP1,CCL23,HD2CM452	HD2Y5W	3	9.56e-01
R148	THP1,CCL23,HD2CM526	HD2Y5W	3	5.55e-01
R148	THP1,CCL23,HD2CM539	HD2Y5W	3	4.22e-02
R86a	THP1	HD2	3	1.10e-04
R86a	THP1,CCL8	HD2	3	9.55e-01
R86a	THP1,CCL8,HD2	HD2	3	NA
R86a	THP1,CCL8,HD2SCR	HD2	3	9.89e-01
R86a	THP1,CCL8,HD2Y5W	HD2	3	2.71e-02
R86a	THP1,CCL8,HD2T6W	HD2	3	2.71e-01
R86a	THP1,CCL8,HD2T6D	HD2	3	1.04e-03
R86a	THP1,CCL8,HD2L11I	HD2	3	9.95e-01
R86a	THP1,CCL8,HD2T12W	HD2	3	9.84e-01
R86a	THP1,CCL8,HD2T16C	HD2	3	9.89e-01
R86a	THP1,CCL8,HD2CM304	HD2	3	1.00e+00
R86a	THP1,CCL8,HD2CM307	HD2	3	8.72e-03
R86a	THP1,CCL8,HD2CM452	HD2	3	9.97e-01
R86a	THP1,CCL8,HD2CM526	HD2	3	5.19e-01
R86a	THP1,CCL8,HD2CM539	HD2	3	1.57e-03
